# Decoding the Rotation Effect: A Retrospective Analysis of Lesion Orientation and Its Impact on Wavelet-Based Radiomics Feature Extraction and Lung Cancer Classification

**DOI:** 10.1007/s10278-025-01520-8

**Published:** 2025-05-06

**Authors:** Lun Matthew Wong, Qi-yong Hemis Ai, Ho Sang Leung, Tifffany Yuen-Tung So, Kuo Feng Hung, Yuet-ting Chan, Ann Dorothy King

**Affiliations:** 1 Department of Imaging and Interventional Radiology, Prince of Wales Hospital, The Chinese University of Hong Kong, LG/F, Cancer Centre, Shatin, New Territory, HKSAR, China; 2 Department of Diagnostic Radiology, School of Clinical Medicine, The University of Hong Kong, HKSAR, China; 3 Department of Imaging and Interventional Radiology, Prince of Wales Hospital, The Hospital Authority - New Territory East Cluster, HKSAR, China; 4 Department of Applied Oral Sciences & Community Dental Care, Faculty of Dentistry, The University of Hong Kong, HKSAR, China

**Keywords:** Radiomics, Wavelet analysis, Reproducibility, Computer tomography, Non-small-cell lung cancer

## Abstract

Wavelet decomposition (WD), widely used in radiomics, redistributes information among derived wavelet components when the input is rotated. This redistribution may alter predictions for the same lesion when scanned at different angles. Despite its potential significance, this vulnerability has frequently been overlooked in radiomic studies while its impact remains poorly understood. Therefore, this study aims to investigate how variations in lesion orientation affect both WD and non-WD radiomic feature values, and subsequently, model performance. We analyzed CT radiomics of primary non-small-cell lung cancer (NSCLC). Prior to feature extraction, we introduced random rotations ranging from 5° to 80° to the tumors. Their effects were quantified by evaluating the percentage difference ($\%\Delta$) between the rotated and unrotated feature values, and validated using Spearman’s rank test. Additionally, radiomics models were trained to discriminate between three histological subtypes of NSCLC using the original features, and then tested on rotated inputs. The correlation between the model accuracies and the degree of rotation was again evaluated using Spearman’s rank test. Four-hundred nineteen NSCLC patients (mean age: 68.1 ± 10.1, 289 men) were evaluated. Significant correlations between feature values and rotations (Spearman’s correlation [CC] magnitude ≥ 0.1, *p* < .05) were found in 23.7% (176/744) of the WD and 0.5% (5/930) of the non-WD texture features. Significant association between performance and rotation was observed in WD-based models built to discriminate between NSCLC histological subtypes (CC =  − 0.44, *p* < .001) but not in non-WD-based models (CC = 0.03, *p* = 0.07). Input lesion orientation affects radiomic feature values and model reproducibility. WD features exhibited significantly greater instability to orientation variations compared to non-WD features.

**Supplementary Information:**

The online version contains supplementary material available at 10.1007/s10278-025-01520-8.

## Introduction

Radiomics is a machine learning technique in radiology that shows promise for the non-invasive characterization of lesions or tissues through extracting and analysing a large quantity of features from a region of interest on medical images [1]. Despite the overwhelming interest in applying deep learning to radiology classification tasks, radiomics remains an active field of research. One of its key advantages is feature transparency with standardized definition [2], which is unmatched by deep artificial neural networks where feature extractors are learned automatically. This transparency facilitates model explainability and interpretability, fostering their linkage with clinical knowledge. A typical component of radiomics workflow is the use of imaging filters to reinforce and segregate image information prior to radiomic feature extraction. In particular, wavelet decomposition (WD) [3], or wavelet transform, is an imaging filter that has gained increasing attention in radiomic studies.

Radiomics studies in the literature generally advocated the inclusion of WD as imaging filters for feature extraction for better model performance [4–8]. However, the literature is less conclusive regarding their repeatability and reproducibility. Most test–retest studies [9–14], with the exception of two [15, 16], indicated inferiority of WD-filters-based features when compared to other features, but they did not proffer an explanation for such inferiority. On the other hand, WD features are similar to or more resilient than non-WD features when there are minor intra- or inter-observer variation in lesion segmentation, provided that the images remain unchanged [13, 17, 18]. This raises questions about the clinical reliability of WD features and underscores the need for careful examination despite their initial benefits.

We postulate that WD features are more susceptible than non-WD features to variations in orientations, as reflected by the results of test–retest studies where maintaining perfect alignment of subject or lesion positioning orientation between tests is often challenging. Additionally, the unique characteristic of WD in splitting the input into multiple complementary components, not seen on other radiomics imaging filters, is also a potential cause of this inferior reproducibility because radiomic models typically selects texture features from only a few WD components, breaking their complementary effect and amplifying variations caused by any orientation differences. Despite efforts to better document these limitations of WD features [2], their drawbacks have been consistently overlooked and underdiscussed in the radiomics community. To our knowledge, only one 2-dimensional (2D) ultrasound study has investigated the effect of different feature extraction angles on WD radiomics model performance [19]. Nonetheless, the level of impact of orientation variations on reproducibility of WD features has never been systematically assessed, especially in 3D images.

Therefore, the purpose of this study is to investigate the effects of variation in lesion orientation on radiomic features, particularly the WD-based features, and also on the radiomic model performance, utilizing an open dataset of non-small-cell lung cancer (NSCLC) patients [20].

## Materials and Method

### Patients

This study retrospectively investigates the open dataset which contains anonymized thorax CT scans of 422 patients (290 men and 132 women; mean age: 67.5; age range: 33–91) with NSCLC previously reported by Aerts et al. collected from Maastricht Radiation Oncology clinic consecutively between May 2005 and July 2010 [20, 21]. The data contains paired CT scans and manual segmentations of the gross tumor volume (GTV) and organs-at-risk, as well as anonymized clinical information including patients’ age, sex, stages, and histology. The histology information was annotated as either “large cell”, “squamous cell carcinoma”, “adenocarcinoma”, “nos” (not otherwise specified) or “NA”. The scans were further examined by a radiologist for exclusion of cases with mis-segmentation. Approval was required by our local institutional review committee, as human data was involved. Approval was obtained with requirement of written consent waived (Ref. 2023.564).

### Image Pre-processing

All images were first intensity normalized using Z-score normalization with the foreground voxels defined by Otsu thresholding. Segmentations of the GTV were extracted by discarding segmentations of other tissues from the raw data. Tiny islands with fewer than 10 connected voxels (roughly 0.01 cm^3^) were considered human errors and removed. After the normalization, we further examined the dimensions of the paired images and segmentation to exclude patients with mismatched images and segmentation. The voxel size and intensity re-binning steps were embedded into the feature extraction pipeline.

### Radiomics Feature Extraction

Radiomic features were extracted using the well-established PyRadiomics (v3.1.0) package [22]. The extraction configuration followed recommended default setting. In brief, images were cropped to the bounding box of the segmentation, resampled into an isometric spacing based on the statistical mode of the dataset, and their intensities were discretized with a fixed bin width prior to the extraction. For imaging filters, 2-dimensional (2D) filters (i.e., 2D local binary pattern) were excluded and wavelet filters were configured with Coif1 wavelets. Eventually, nine imaging filters, including WD, were applied, resulting in the extraction of 93 handcrafted features from each processed image as well as the original image. This yielded a total of 1674 features per patient, comprising 744 WD features and 930 non-WD features. Note that some imaging filters resulted in multiple product images. Details of the radiomic feature extraction configuration are given in the Supplementary material (Appendix A).

### Augmenting GTVs into Different Orientations

Images of the GTVs were augmented together with their paired segmentation by rotating the GTV about its centre of mass, the rotations were sampled randomly from normal distributions with means from 5° to 80° at 5° intervals, and a uniform standard deviation of 10°. One set of such rotation matrices were generated for each GTV. These rotations represent ascending levels of deviation from the original orientations (${R}_{0}$). Radiomic features were then extracted from the original and rotated images and grouped by the rotated degrees as ${R}_{i}, i\in \{0, 5, 10, \dots , 80\}$. Technical details of how these angles were statistically sampled are further reported in the Supplementary material (Appendix A).

Prior to feature extraction, rotational matrices were applied by resampling the image. To minimize the bias arising from the resampling process, ${R}_{0}$ also underwent resampling but without applying any rotations.

### Evaluating Effects of the Rotations

Two levels of analyses were performed to investigate radiomic features repeatability: first, by evaluating how their numerical values respond to changes in orientation (feature-level analysis); and second, by evaluating how the performance of a radiomic model trained using ${R}_{0}$ features for prediction of NSCLC histological subtypes changed when rotated features (${R}_{5}$ to ${R}_{80}$) were used (performance-level analysis).

#### Measuring Stability of Feature Values against Orientation Variations

The deviation in the feature values extracted from images before (${R}_{0}$) and after the application of rotations (${R}_{05}$ to ${R}_{80}$) were measured by the percentage difference ($\%\Delta$) considering the diverse value ranges of radiomic features. Higher $\%\Delta$ indicates greater deviation from the original un-rotated feature. The association of $\%\Delta$ and the rotation degrees introduced was studied using Spearman’s rank test, where a significant *p*-value (*p* < 0.05) and a Spearman’s correlation coefficient (CC) magnitude ≥ 0.1 indicated the feature presented correlations with rotation degrees with a non-trivial effect size—a metric that indicates the strength of statistically significant effects [23]. In this study, the effect sizes of Spearman’s rank test were stratified as trivial (|CC|< 0.1), weak (0.1 ≤|CC|< 0.3), moderate (0.3 ≤|CC|< 0.7) and strong (|CC|≥ 0.7). An ideal, reliable feature should not be correlated to the orientation of tumor (Spearman’s *p*-value being insignificant close to 1 and CC close to 0). Additionally, since $\%\Delta$ is signed, such that positive $\%\Delta$ and negative $\%\Delta$ of the same feature in different patients could cancel out, the inter-quartile-range (IQR) was also used to measure the magnitude of the discrepancy induced by the rotations, with a wider IQR indicating that the feature was more unstable and less repeatable when subjected to input rotations.

Subgroup analysis was then conducted by grouping the results with respect to the imaging filters (e.g., original, exponential, wavelets…etc.) and the texture categories (e.g., first order, GLCM, GLRLM…etc.), following the hierarchy of the well-established radiomics feature extraction software Pyradiomics (v3.1.0) [22].

#### Measuring Stability of Model Performance against Orientations Variations

We tested the ability of radiomic models built from feature groups ${X}_{WD}$ and ${X}_{nWD}$ individually to discriminate NSCLC primary tumor into three histological subtypes of (i) large cell carcinoma (LCC), (ii) squamous cell carcinoma (SCC), and (iii) adenocarcinoma (ADC). Performance of the models were evaluated using conventional accuracy as the primary metric, and macro average of f1-score, sensitivity and specificity evaluated in one-versus-rest manner. To mitigate sampling bias and enabled statistical analysis, we conducted repeated fivefold cross-validation 50 times, each shuffled the patients randomly before splitting them into 5 folds (a total of 250 train-test cycles). In each cross-validation, radiomic models were trained using ${R}_{0}$ features of patients in 4 training folds, and then tested using ${R}_{0}$ to ${R}_{80}$ features of patients in the remaining testing fold. This configuration simulates the situation where the trained diagnostic model encounter lesions with various degree of scan or grow orientation. The radiomic training and testing pipeline is reported in the next section.

**Fig. 1:**
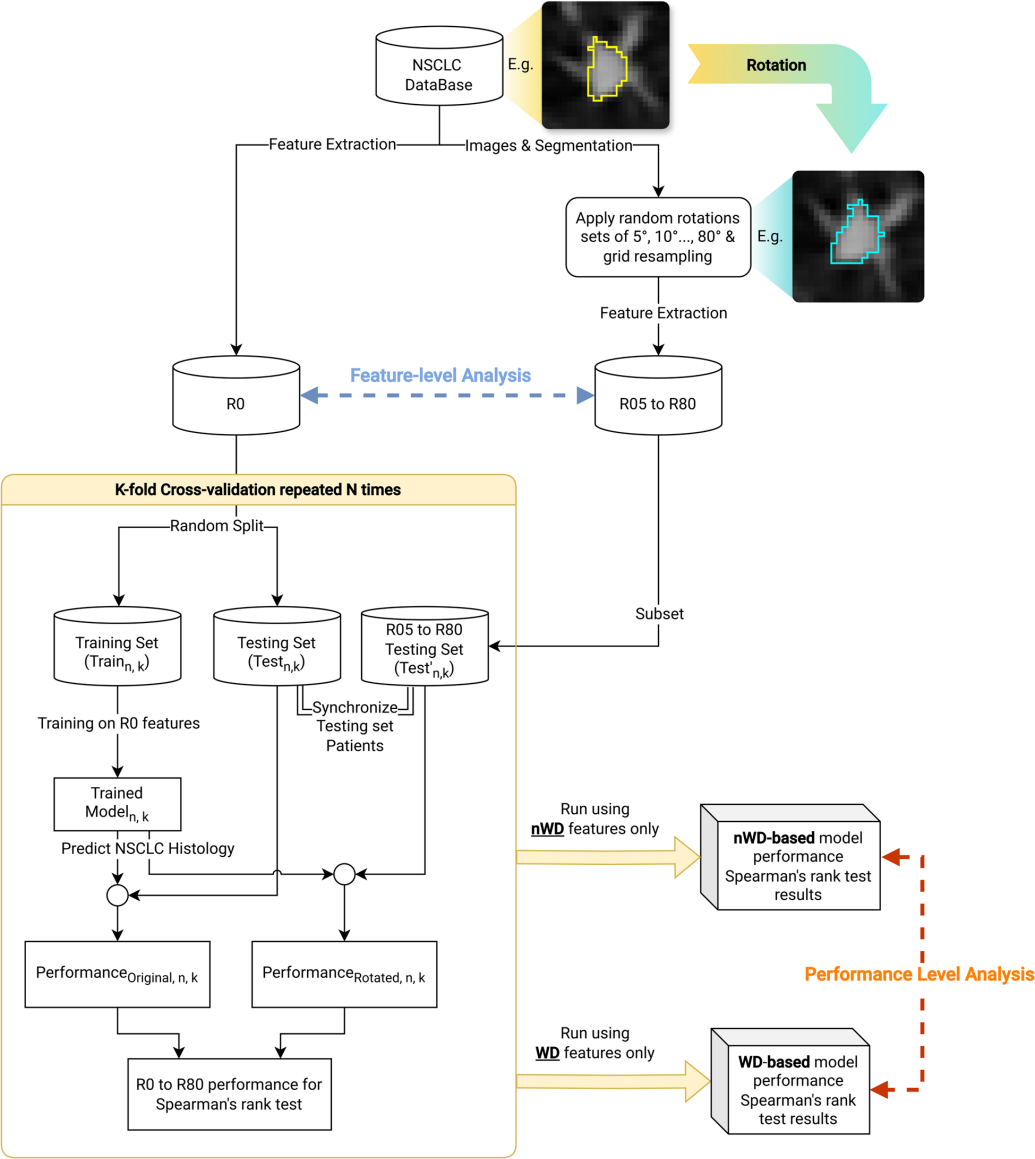
Flowchart of the repeated K-Fold cross-validation study design. The process was executed independently for wavelet-decomposition (WD) and non-wavelet-decomposition (nWD) feature sets, with subsequent comparative analysis of the outcomes. For each feature set, two main statistical analyses were conducted: (1) determination of whether feature values derived from a lesion were affected by the orientation of scanning grid; and (2) evaluation of the stability of radiomic model performance in differentiating non-small-cell lung cancer subtypes when trained on the original model and tested against rotated input data. The study parameters were set at N = 50 subjects and K = 5 folds for cross-validation. CV = cross-validation, WD = wavelet decomposition, nWD = non-WD, NSCLC = non-small-cell lung cancer

After all the runs were completed, performances of the trained model in predicting NSCLC histological subtypes were evaluated to investigate how deviation of GTV imaging orientation $({R}_{0}$ to ${R}_{80}$) from the training orientation (${R}_{0}$) could affect model performance. Similar to feature-level analysed, Spearman’s rank test was used to identify whether a statistically significant correlation between histology discrimination performance and applied rotation existed, as well as their effect sizes [23]. Comparisons were further made between the results from ${X}_{WD}$ and ${X}_{nWD}$. A flowchart depicting the study flow is provided in Fig. 1.

### Radiomics Model Building Pipeline

A standard radiomic model building pipeline was adopted to train machine learning models to discriminate the segmented lung cancers GTV histological subtypes. Patients with subtypes labelled as “not otherwise specified” (NOS) or “N/A” (no histology information) were excluded from this part of the study to prevent the unclear histological data from confounding the observed effects with those of WD features. The radiomics pipeline comprised three phases: (i) the preliminary feature filtering phase based on all samples, (ii) the fine feature selection phase based on cross-validation training set, and (iii) model building phases testing 5 classification models in parallel. The training of this pipeline involved ${R}_{0}$ features only, the design principle here was to train radiomics models using original, non-rotated ${R}_{0}$ features, and then tested the trained model with all ${R}_{0}$ to ${R}_{80}$ features to evaluate their performance in discriminating NSCLC histology, hence, evaluating the effects of lesion orientation discrepancies. Additional technical detail of the implementation of this pipeline is provided in the Supplementary material (Appendix A).

### Statistical Analysis Software

Confidence intervals were estimated based on Student’s standardized distribution which reference the variable mean and standard deviation. All statistical tests were performed using either python alone, with opensource package “scipy.stat” (v1.10.1), or the combination of python and SPSS v28 (IBM, Armonk, USA) through SPSS’s native application interface.

### Data Availability Statement

The original data utilized is released by Aerts et al. under the license CC-BY-NC 3.0 [20]. The data generated from this study, which includes the radiomic features extracted from the original (${R}_{0}$) and the rotated $({R}_{5}$ to ${R}_{80}$) GTV are available on request. The source code used in this study is publicly available on an online repository (https://github.com/ml-w/wavelets_n_rotation).

## Results

### Patients

**Fig. 2:**
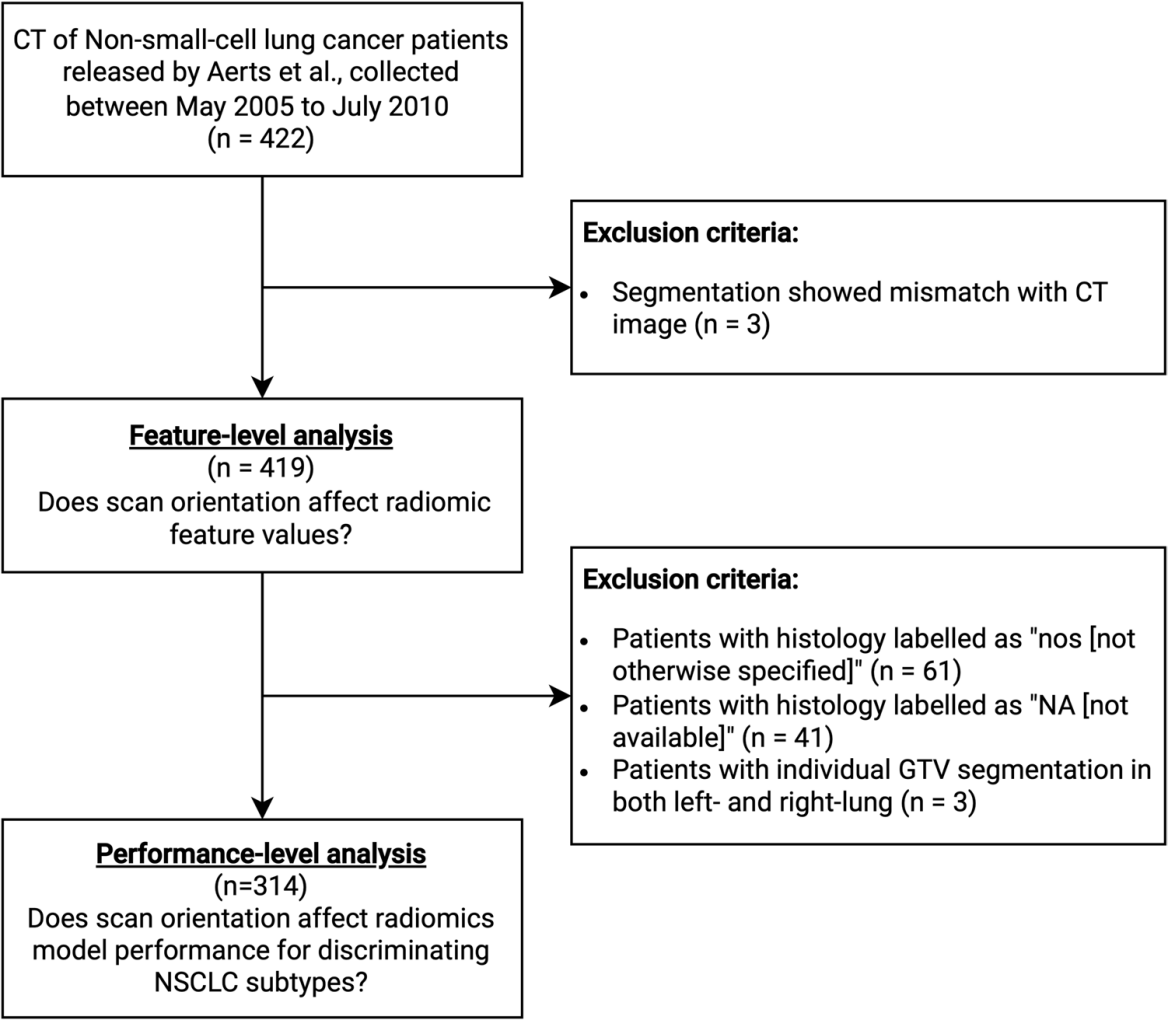
Study flow diagram. This study utilized public data of non-small-cell lung cancer (NSCLC) patients released by Aerts et al. [20] who underwent CT examination with paired tumor segmentation. GTV = gross tumor volume, nos = not otherwise specified, NA = not available

For the first part of the study to evaluate the robustness of WD features on the feature level, 3 patients out of 422 were excluded from all analysis because of the mismatched CT and segmentation spatial alignment, leaving 419 patients (mean age: 68.1 ± 10.1, 289 men). For the second part of the study, evaluating the classification performance of the radiomics model, 61 and 41 patients with histology marked as “NOS” and “N/A”, respectively, were further excluded. In addition, we further excluded 3 more patients with individual tumor segmentations on both sides of the lung, which confused the interpretation of the reported histology, leaving 314 patients (Fig. 2).

Additional rationales regarding excluded cases are reported in the Supplementary material (Appendix A). Patient characteristics for each level of analysis are tabulated in Table 1:

**Table 1:** Patents characteristics of the retrospectively studied non-small-cell lung (NSCLC) cancer patients

Variable	Original dataset (*n* = 422) Frequency (%)	Phase 1 (*n* = 419) Frequency (%)	Phase 2 (*n* = 314) Frequency (%)
**Gender**			
Male	68.7 (290/422)	69.0 (289/419)	67.8 (213/314)
Female	31.3 (132/422)	31.0 (130/419)	32.2 (101/314)
**Histology**			
Large cell carcinoma	27.0 (114/422)	27.2 (114/419)	36.3 (114/314)
Squamous cell carcinoma	36.0 (152/422)	36.3 (152/419)	47.5 (149/314)
Adenocarcinoma	12.1 (51/422)	12.2 (51/419)	16.2 (51/316)
nos	14.9 (63/422)	14.6 (61/419)	0 (0/314)
NA	10.0 (42/422)	9.8 (41/419)	0 (0/314)
**T-stage**			
T1	22.0 (93/422)	22.2 (93/419)	16.9 (53/314)
T2	37.0 (156/422)	36.8 (154/419)	39.2 (123/314)
T3	12.6 (53/422)	12.4 (52/419)	13.7 (43/314)
T4	27.7 (117/422)	27.9 (117/419)	29.9 (94/314)
Tx	0.5 (2/422)	0.5 (2/419)	0.3 (1/314)
**N-stage**			
N0	40.3 (170/422)	40.3 (169/419)	35.7 (112/314)
N1	5.5 (23/422)	5.3 (22/419)	6.7 (21/314)
N2	33.4 (141/422)	33.7 (141/419)	35.7 (112/314)
N3	20.1 (85/422)	20.0 (84/419)	21.0 (66/314)
Nx	0.7 (3/422)	0.7 (3/419)	1.0 (3/314)
**TNM stage grouping**			
Stage I	22.0 (93/422)	22.0 (92/419)	15.6 (49/314)
Stage II	9.5 (40/422)	9.5 (40/419)	11.1 (35/314)
Stage IIIa	26.5 (112/422)	26.5 (111/419)	29.3 (92/314)
Stage IIIb	41.7 (176/422)	41.8 (175/419)	43.6 (137/314)

*Tx*, tumor stage is inconclusive; *Nx*, node stage is inconclusive; *nos*, not otherwise specified

### Feature-Level Analysis: Does Scan Orientation Affect Radiomic Feature Values?

#### Shape Features

As shape features are independent on the imaging filter in PyRadiomics, they were separately analysed. Most, 11 out of 14, shape features showed good robustness without statistical significance in Spearman’s correlation (all |CC|< 0.01, *p* > 0.05) against different degrees of rotation. The three remaining features were 2D features, namely Maximum 2D Diameter Column, Row and Slice. Detailed results from Spearman’s analysis are reported in Supplementary material (Table S1, Appendix B).

#### Texture Features

Comparing the effects of input orientations among imaging filters, 7 out of 8 WD components (except LLL, with IQR of $\%\Delta$ = 3.2% [95% CI: 3.0%–3.4%]) showed significant changes in feature values, reflected by the wide $\%\Delta$ IQR ranging from 14.5% to 48.0%. They also had large portion of features (up to 53.76% [50/93] in LLH) with a statistically significant, non-trivial correlation between $\%\Delta$ and the magnitude of rotation degree from Spearman’s rank test (*p* < 0.05 and |CC|≥ 0.1).

Conversely for non-WD features, with the exception of the “square” imaging filter (IQR in $\%\Delta$=23.3%), the original and all the other 6 non-WD filtered images showed a narrow IQR in $\%\Delta$ ranging from 3.0% to 7.0%. Also, with the exception of origin (1.08% [1/93]) and LBP_3D-m2_ (4.30% [4/93] features with non-trivial correlation), all the other 6 imaging filters had none of the derived texture features showing a significant correlation with between $\%\Delta$ and magnitude of rotation applied.

**Fig. 3:**
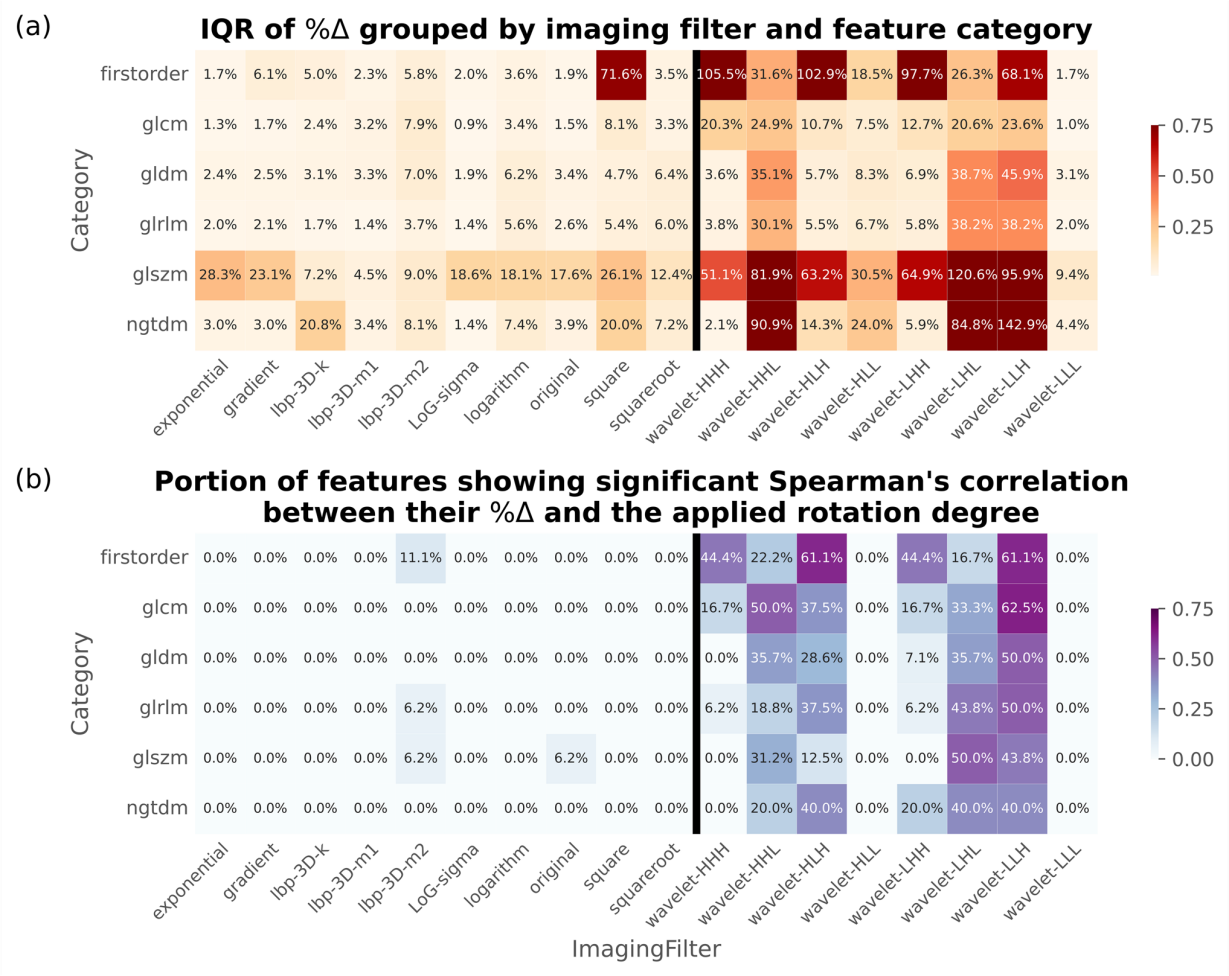
Heatmaps visualizing the distribution of radiomic feature values before (${R}_{0}$) and after rotation (${R}_{05}$ to ${R}_{80}$), grouped by the imaging filters and the feature categories, the values displayed are the mean across all rotation degrees. Numerical values are (a) IQR of percentage differences ($\%\Delta$) and (b) percentage of features with a significant correlation coefficient with rotated degrees (defined as absolute Spearman’s coefficient ≥ 0.1 between the feature values and the orientation differences). Darker cells represent a more severe influence by orientation differences. These plots demonstrate the inferior stability in wavelet decomposition (WD) features (right side of the black vertical separator) when compared to non-WD features (left side). GLCM = gray-level co-occurrence matrix, GLDM = gray-level dependence matrix, GLRLM = gray-level run length matrix, GLSZM = gray-level size zone matrix, H = high-pass filter, IQR = interquartile-range, L = low-pass filter, lbp = local binary pattern, log = Laplacian of Gaussian, NGTDM = neighbourhood gray tone difference matrix

**Fig. 4:**
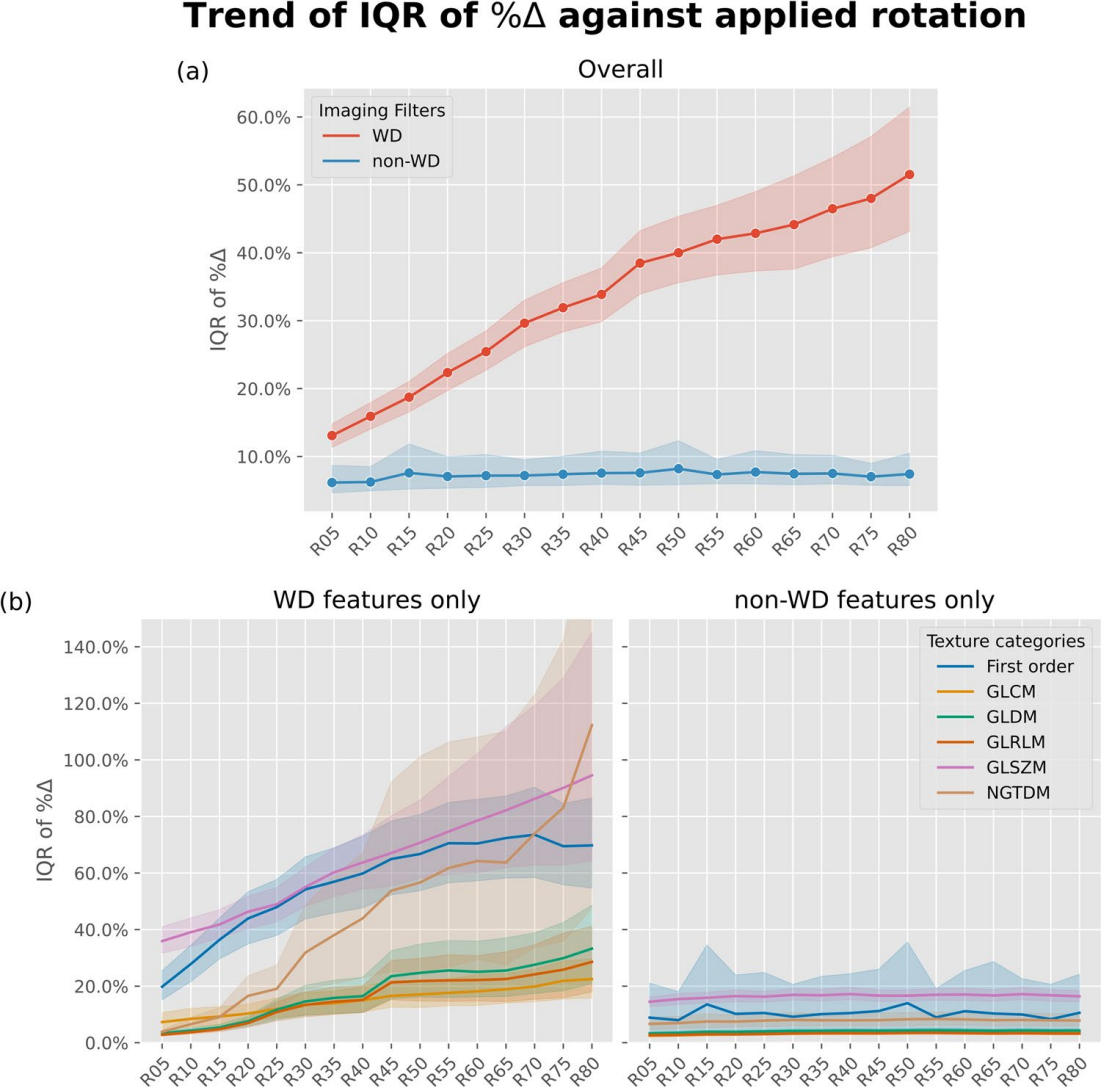
Plots of inter-quartile-range (IQR) of percentage differences ($\mathrm{\%}\Delta$) in radiomic feature values before and after applying various degrees of rotation. The plot (a) overviews of comparison between WD and non-WD features, and (b) compares WD and non-WD features grouped by texture categories, the two plots share the same y-axis. WD features exhibit more vigorous changes in IQR of $\mathrm{\%}\Delta$ compared to non-WD features. The transparent bands represent the 95% confidence interval of taking the measurements of IQR of $\mathrm{\%}\Delta$. The x-axes represent rotation sets on all plots, from a mean of 5 degrees (R05) deviation from original orientation to a mean of 80 degrees (R80). GLCM = gray-level co-occurrence matrix, GLDM = gray-level dependence matrix, GLRLM = gray-level run-length matrix, GLSZM = gray-level zone size matrix, IQR = inter-quartile range, NGTDM = neighbourhood ray-tone differences matrix, WD = wavelet decomposition

These results are visualized in a heatmap (Fig. 3) and line plot (Fig. 4). Detailed numerical results are tabulated in the Supplementary table (Table S1 and S2, Appendix B). In addition, the mean distribution of IQR of $\%\Delta$ of each radiomic features across different degrees of rotation, grouped by different imaging filters (including shape features), are provided in box plots in the Supplementary material (Appendix C).

### Performance-Level Analysis: Does Scan Orientation Affect Radiomics Model Performance for Discriminating NSCLC Subtypes?

Regarding feature selection phases, which refers to the two phases in the radiomics models building pipeline (i) preliminary feature selection, and (ii) fine feature selection phase, the WD-based models, denoted as ${M}_{WD}$, incorporated an average of 24.8 (range: 20–29) out of 744 WD features, and the non-WD models ${M}_{nWD}$ incorporated an average of 21.1 (range: 17–20) out of 930 non-WD features. Both WD and non-WD models were only trained using ${R}_{0}$ unrotated features.

The trained ${M}_{WD}$ yielded the highest performance when tested with unrotated ${R}_{0}$ features compared to the corresponding ${M}_{nWD}$ at accuracies of 49.9% ± 2.0% and 49.0% ± 2.7% (*p* < 0.05), respectively. However, when these trained models were applied to predict NSCLC subtypes using features extracted from tumor segmentation rotated by 5° to 80° (${R}_{05}$ to ${R}_{80}$) instead, the performance of ${M}_{WD}$ models exhibited a declining trend with increasing applied rotation (*p* < 0.001 and |CC| ranged from 0.40 to 0.57), whereas ${M}_{nWD}$ showed insignificant trend, except on logistic regression and k-nearest-neighbours, the later showed a positive but small CC of 0.113 (Table 2). Similar trend was observed for other performance metrics including f1-score, sensitivity and specificity, with all ${M}_{WD}$ showing a significant decrease trend (all *p* < 0.05, |CC| ranged from 0.09 to 0.573), while ${M}_{nWD}$ showing an insignificant trend except for logistic regression and k-nearest-neighbour with a small effect size (|CC| ranged from 0.02 to 0.113) (Table 3). The top ten performing folds showed an accuracy of 68.3% to 69.8%, three of which were ${M}_{WD}$.

**Table 2:** Table of statistical analysis of the association between model performance and rotated degrees (${R}_{0}$ to ${R}_{80}$). All of the models built with WD-based features showed significant trends with a moderate effect size (0.3 ≤|CC|< 0.7, *p* <.05). On the other hand, only one in five classifiers built from non-WD-based features, ${M}_{nWD, KNN}$ resulted in a significant correlation with rotated degrees

Classifiers	Spearman’s CC [95% Confidence Interval]	Spearman’s *p*
**WD-based models (*n* = 1250)**		
**All**	− 0.436^a^ [− 0.46, − 0.41]^a^	<.001*
KNN (*n* = 250)	− 0.457^a^ [− 0.51, − 0.40]^a^	<.001*
Logistic Regression (*n* = 250)	− 0.573^a^ [− 0.62, − 0.53]^a^	<.001*
Random Forest (*n* = 250)	− 0.388^a^ [− 0.44, − 0.33]^a^	<.001*
Support Vector Machine (*n* = 250)	− 0.494^a^ [− 0.54, − 0.44]^a^	<.001*
Support Vector Machine (rbf) (*n* = 250)	− 0.401^a^ [− 0.46, − 0.34]^a^	<.001*
**nWD-based models (*n* = 1250)**		
**All**	0.028 [− 0.0, 0.06]	.068
KNN (*n* = 250)	0.113 [0.05, 0.18] ^a^	<.001*
Logistic Regression (*n* = 250)	0.074 [0.01, 0.14]	.031*
Random Forest (*n* = 250)	− 0.031 [− 0.10, 0.04]	.363
Support Vector Machine (*n* = 250)	0.037 [− 0.03, 0.1]	.284
Support Vector Machine (rbf) (*n* = 250)	0.023 [− 0.04, 0.09]	.512

*Marks *p*-values <.05; ^a^ Marks moderate effect size with a Spearman’s CC magnitude ≥ 0.1

*WD*, wavelet decomposition; *nWD*, non-WD; *CC*, correlation coefficient; *KNN*, K-nearest-neighbours; *rbf*, radial basis function

**Table 3:** Table of statistical analysis of the association between model performance and rotated degrees (${R}_{0}$ to ${R}_{80}$) grouped by different performance metrics. Similar trend as in Table 2 was observed

Performance metrics	Spearman’s CC [95% Confidence Interval]	Spearman’s *p*
**WD-based models (All *n* = 1250)**		
Accuracy	− 0.436 [− 0.46, − 0.41]^a^	<.001*
F1-score	− 0.310 [− 0.34, − 0.28] ^a^	<.001*
Sensitivity	− 0.355 [− 0.38, − 0.33] ^a^	<.001*
Specificity	− 0.182 [− 0.21, − 0.15] ^a^	<.001*
**nWD-based models (All *n* = 1250)**		
Accuracy	0.028 [− 0.00, 0.06]	0.068
F1-score	0.012 [− 0.02, 0.04]	0.435
Sensitivity	0.024 [− 0.01, 0.05]	0.123
Accuracy	0.002 [− 0.03, 0.03]	0.901

*Marks *p*-values <.05; ^a^ Marks moderate effect size with a Spearman’s CC magnitude ≥ 0.1

WD, wavelet decomposition; *nWD*, non-WD; *CC*, correlation coefficient

**Fig. 5:**
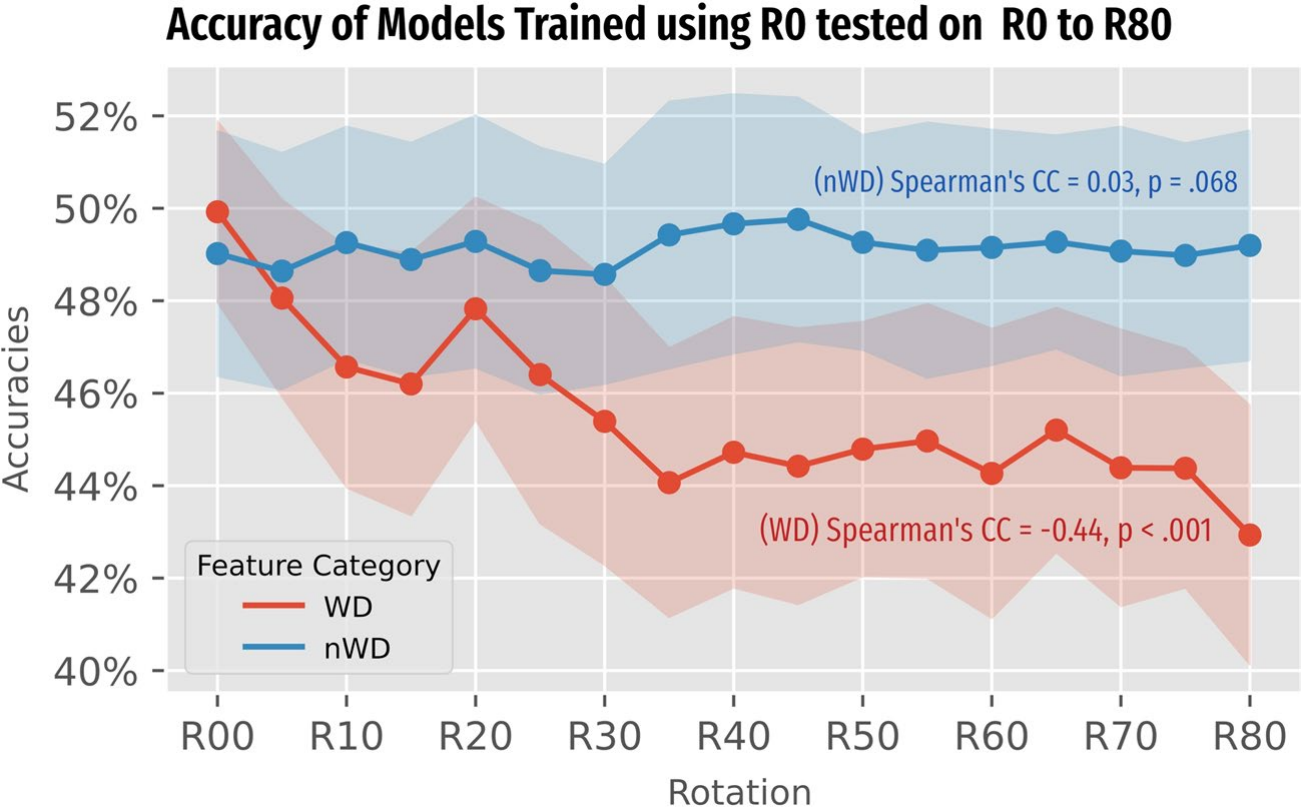
Plots of accuracies from 50 repetitions of fivefold cross-validation of radiomic models to discriminate three major NSCLC subtypes. The transparent bands represent the ± 1 standard deviation band around that measures spread of performance across cross-validations. WD models showed a general decline with rotation degree from Spearman’s analysis, exhibiting a moderate effect size, whereas non-WD models did not suffer from such decline with a trivial effect size from Spearman’s analysis, as well as a non-significant *p*-value (*p* =.068). The x-axis rotation sets on all plots, from the original image set (R00) to a set with mean 80 degrees orientation difference to the original image (R80). NSCLC = non-small-cell lung cancer, WD = wavelet-decomposition, nWD = non-WD, KNN = K-nearest neighbours, GBC = gradient boost classifier, ML = machine learning

The performance across different rotation sets is plotted in Fig. 5. The detailed performance of each classifier models is reported in the Supplementary material (Table S4, Appendix B). Further evaluation of metrics other than accuracies, including f1-score, sensitivity and specificity were evaluated and reported as well (Table S5, Appendix B). Additionally, the frequencies of the radiomic features being selected during fivefold cross-validation is also reported (Table S6 and S7, Appendix B).

## Discussion

In this study, we demonstrated that WD features are statistically significantly more susceptible than non-WD features to orientation variations, this significantly affects not only the test–retest reproducibility of the feature values, but also introduces instability in the performance of the models built. This implies varying orientation alone can potentially lead to a completely different conclusion by the radiomics model. In particular, we showed 7 out of 8 WD components exhibited various degrees of discrepancies before and after the application of rotation, reflected by greater IQR of $\%\Delta$ (WD: 34.0%[CI:32.7%–35.4%]; non-WD: 7.3%[CI:6.6%–7.9%]) and higher portions of features with significant value changes across different rotations (WD: 22.7% [176/744]; non-WD: 0.5% [5/930]). While this effect of orientation appears to be quite linear on the feature value level, it was much more complicated for the radiomics models built. Although similar observation of a decline in performance was observed for all WD-based classifier model for discriminating NSCLC histology, the trend was not monotonic and rose momentarily at ${R}_{20}$, indicating the classifier model has some tolerance to noise arising from orientation variations of target lesion. Additional interpretation and discussion of specific patterns seen on each feature against rotation is available in the Supplementary material (Appendix D).

Our results suggest that extra caution should be exercised regarding the inclusion of WD features. The decision to include these features must be carefully examined based on the specific characteristics of the lesion in query. Excluding WD filters is advisable when extracting radiomics features from lesions without a reference morphology that allows for orientation standardization, including NSCLC. Furthermore, inclusion decision should be accompanied by methodologies to aggregate features of the same texture descriptor from all the WD components to prevent information leaking into unselected WD components (those that were filtered away during feature selection step) due to orientation variations. Ideally, future research should adopt WD kernels or mother wavelets that generate rotationally invariant components to harness the advantage of WD without compromising reproducibility and clinical intuitiveness.

Interestingly, the “LLL” WD component (i.e., the component with low-pass filter applied to all three axis) showed much more stable and resilient feature values compared to other WD components. This pattern is also consistent with results reported in several previous work, where majority of WD components except the LLL component exhibited inferior repeatability in a test–retest setup [11, 14, 15, 24], but these studies did not discuss nor proffer an explanation for it. It is likely because, depending on the mother wavelet form used, the “LLL” component is generally equivalent to the under-sampled version of the original image and has little effect in enhancing directional information that are susceptible to the effects of rotations. However, that also suggests the LLL component might not have much value as under-sampling typically contains less information as the original image.

While our primary interest lies in understanding how the performance was impacted by GTV orientation variations between training and inference data, we acknowledge that the baseline performance of the trained model on ${R}_{0}$ inference data was suboptimal at 49.9% ± 2.0% and 49.0 ± 2.7% for ${M}_{WD}$ and ${M}_{nWD}$, respectively. However, a critical weakness of majority of existing studies which reported better results, including those that discriminated only between ADC and SCC [25–28] and between all four subtypes [29–31], is that they have adopted a fixed train-test split or only performed a single K-fold cross-validation that can subject to selection bias, as opposed to our approach of repeating fivefold cross-validation 50 times and drawing the average. Only one study in the literature investigated this problem with a nested K-fold setting similar to our repeated K-fold approach [29]. The authors reported that for 3-class discrimination between ADC, SCC and LCC, identical to our setting, their radiomics pipeline attained an accuracy of 53.0% ± 5.6%, validated on multiple NSCLC public dataset including the one we used. This accuracy, though slightly better, was consistent with our observations given they did not separately evaluate WD and non-WD features. In this regard, further improvement of model performance might require an expansion of the training dataset to achieve and validate.

Nevertheless, there are some limitations in this study. First, the random sampling of rotations did not cover all possible orientations because we only defined them by two rotation actions but there are three degrees of freedom. Second, this study only accounts for 3D image radiomics, and the results could not be casually extended to 2D imaging modalities like ultrasound and X-ray. Third, this study has only looked into one mother wavelet, the first order Coiflets wavelet (“Coif1”), which is the default setting recommend by the well-established package PyRadiomics [22]. The rationale of this decision was to maximize the applicability of the results through testing the utilized by choosing a representative mother wavelet that is commonly used. In particular, majority of the lung radiomics studies reviewed have utilized the Coif1 mother wavelet (details available in Supplementary material – Appendix A). Nonetheless, our findings can likely extend to separable mother wavelets that shares the lack of rotational invariance and are frequently used in radiomics [2]. This highlights the needs for future radiomics studies to consider rotationally invariant wavelet transform [32, 33], and explore WD specific features that are less susceptible to input orientations changes [34]. Forth, we cannot fully exclude the partial volume effect during image resampling which was necessary to introduce rotations to the images. Finally, we have only conducted the test on one modality and one disease.

## Conclusion

We revealed orientation of lesion significantly impacted more CT WD features than non-WD features in NSCLC patients. Variations in tumor orientation or scan position alone can alter the prediction of a trained WD-based model. The inclusion of WD features in radiomics model building should be approached with caution, especially where there is little control over the growth orientation of a lesion. Future radiomics studies should explore WD algorithms with rotational invariant property to improve the reliability of radiomics lesion characterization models.

## Supplementary Information

Below is the link to the electronic supplementary material.

Supplementary file1 (DOCX 1899 KB)

Supplementary file2 (DOCX 48 KB)

Supplementary file3 (DOCX 12278 KB)

Supplementary file4 (DOCX 21 KB)

## Data Availability

The dataset analysed in this study is publicly available, and the derived data, including the extracted radiomic features, can be obtained from the first author upon reasonable request.

## Abbreviations

%Δ: Percentage difference
2D: Two-dimensional
ADC: Adenocarcinoma
CC: Spearman’s correlation coefficient
GTV: Gross tumor volume
H: High-pass filter
IQR: Inter-quartile-range
L: Low-pass filter
LCC: Large cell carcinoma
NOS: Not otherwise specified
nWD: Non-Wavelet-Decomposition
NSCLC: Non-small-cell lung cancer
SCC: Squamous cell carcinoma
WD: Wavelet Decomposition
