# Supplementary material for: Decoding the Rotation Effect: A Retrospective Analysis of Lesion Orientation and Its Impact on Wavelet-Based Radiomics Feature Extraction and Lung Cancer Classification
Source: J Imaging Inform Med. 2025 May 6;39(1):265–76. doi: 10.1007/s10278-025-01520-8 (PMC12920822; doi:10.1007/s10278-025-01520-8)
Supplement: Supplementary file 1 — Supplementary file1 (DOCX 1899 KB) [file 10278_2025_1520_MOESM1_ESM.docx]

Supplementary material - Appendix A: Detail Methodology

# Methods and Materials

## Details of radiomics features extraction

### Imaging pre-processing

Prior to feature extraction, we performed image intensity normalization to harmonize any potential batch effects that might exist within the scanned CT images. Normalization was done using an in-house developed open-source package written in Python, available online [1], which is based-on image processing library SimpleITK . Although this package was originally written for the normalization of MRI images, the normalization pipeline can be flexibly customized to cater for other modalities as well. Using this package, we construct a normalization graph which generates the foreground mask using the Otsu thresholding method, followed by Z-score normalization that shifts the intensity distribution within the mask linearly to unit variance and zero mean.

The GTV segmentations were also pre-processed to remove human errors and identify those with multiple GTV segmentations. Specifically, we computed the number of connected components in each GTV segmentation and reviewed those who have more than one GTV masses labelled. Tiny components with less than 10 connected voxels (roughly 0.01cm^3^) was considered as human errors and discarded. Otherwise, each of the connected component was considered an individual sample during later radiomic feature extraction step and saved as a cropped image patch defined by a bounding box that encapsulates it with a 1 cm padding.

Finally, each cropped image patch was further resampled to isotropic spacing with a size of (0.98mm × 0.98mm × 0.98mm).

### Feature extraction

In this study, radiomics features were extracted using the well-established Python package PyRadiomics (v3.0.0) [2]. This package provides a wide ranges of common 2D/3D imaging filters and feature descriptors that are defined in the Image Biomarker Standardization Initiative (IBSI) list. Typically, the pre-processed input image will be further processed by an imaging filter, followed by re-binning the derived image intensities to a fixed bin-width of 0.5 (note that the derived image does not always have a standard physical unit). Then, up to 94 texture features can be extracted, with “Standard Deviation” disabled by default because of its obvious correlation with “Variance”, leaving 93 features per imaging filter in this study. A complete list of this 94 texture features is available online the official documentation [3] and is not repeated in this material for readability.

PyRadiomics provides nine imaging filters: Wavelet Decomposition (WD), Laplacian of Gaussian (LoG), Square, Square Root, Logarithm, Exponential, Gradient, Local Binary Pattern 2D (LBP_2D_), and LBP 3D (LBP_3D_). Given our study's focus on 3D modality CT, we excluded LBP_2D_, which focuses on 2D features. All other filters were employed for feature extraction. WD with “Coif1” mother wavelet was selected to enhance the representativeness of this study considering it is the default setting of the well-established feature extraction package Pyradiomics. It is also the most utilized WD mother wavelet used in all cited lung radiomics studies.

Table-SA 1 Summary of the type of mother wavelet used in lung radiomics studies reviewed. Majority of the studies that applied WD utilized Coif1 as mother wavelet. N/A = WD was not applied, ? = WD applied but type of mother wavelet not mentioned.

| Reference # | **Type of mother wavelet used** |
| --- | --- |
| [6] | Coif1 |
| [21] | Coif1 |
| [25] | Coif1 |
| [26] | ? |
| [27] | Coif1 |
| [28] | N/A |
| [29] | Haar |
| [30] | Coif1 |
| [31] | ? |

Notably, all these filters only generate a single filtered image, with the exceptions of WD and LBP_3D_. By default, the LBP_3D_ filter calculates two levels of spherical harmonics (LBP_3D-m1/m2_) and an additional kurtosis image (LBP_3D-k_), generating three filtered images per input image. Meanwhile, the WD yields eight WD components corresponding to the permutation of high- and low-pass filters individually applied along the x-, y-, and z-axis of the 3D Cartesian grid for each input image. It is worth highlighting that LBP_3D_'s implementation used in this study exploited spherical harmonics to construct a rotationally invariant descriptor, theoretically unaffected by orientation of inputs [4], whereas WD does not have such rotational invariant property.

We adopted the typical pipeline for feature extraction. First, each image was filtered into an additional 17 images (8 from WD, 3 from LBP_3D_, 6 from the other filters). Then, from each original and filtered image (total of 18), we extracted 93 features, resulting in a total of 1674 texture features. Finally, independent to imaging filters, we also extracted 14 shape features for each segmented tumor. The YAML setting file for feature extraction is also available in the project repository.

## Details of augmenting gross tumour volumes (GTVs) into different orientations

### Sampling rotation matrix

Transform matrices were sampled randomly for each lesion with the aim to rotate the whole dataset on average 5⁰ to 80⁰ degrees with a 5⁰ interval relative to the axial axis. This was achieved by sampling a first yaw (rotation about axial axis) from the normal distribution with a mean of the desired rotation degree, with the standard deviation empirically set to 10⁰. Then sampling and a pitch rotation (rotation about sagittal axis) from a the normal distribution with a mean of 0 and a standard deviation of the desired rotation degree (Figure S1).

After sampling the yaw and pitch degrees, a rotation matrix was constructed following the definition of the Euler angles. For example, to introduce a yaw $\alpha$ and then a pitch $\beta$, the rotational matrix is defined by:

$$R(\beta, \alpha) = R_{y}\left( \beta\right)R_{z}\left( \alpha\right)$$

$$\mathrm{where} R_{y}\left( \beta\right)=\left[ \begin{matrix} \cos\beta& 0 & \sin\beta\\ 0 & 1 & 0 \\ -\sin\beta& 0 & \cos\beta\end{matrix} \right], R_{z}\left( \alpha\right)=\left[ \begin{matrix} cos\alpha& -\sin\alpha& 0 \\ \sin\alpha& \cos\alpha& 0 \\ 0 & 0 & 1 \end{matrix} \right]$$

This matrix rotates the image about the origin. To achieve rotation about the lesion centre, the coordinate of the lesions’ centre of masses (COM) were calculated based on the segmentation, and then the whole image was shifted to align the COM with the origin prior to applying the rotational matrix. The inversed shift was then applied to transform the COM back to its original position. The whole transformation can be written as follow.

$$\vec{r}'=R(\beta,\alpha)\left( \vec{r}-\vec{b} \right)+\vec{b}$$

Where $\vec{b}$, $\vec{r}$ and $\vec{r}'$ are coordinate vectors of the original segmentation COM, the original image grid and the rotated image grid, respectively.


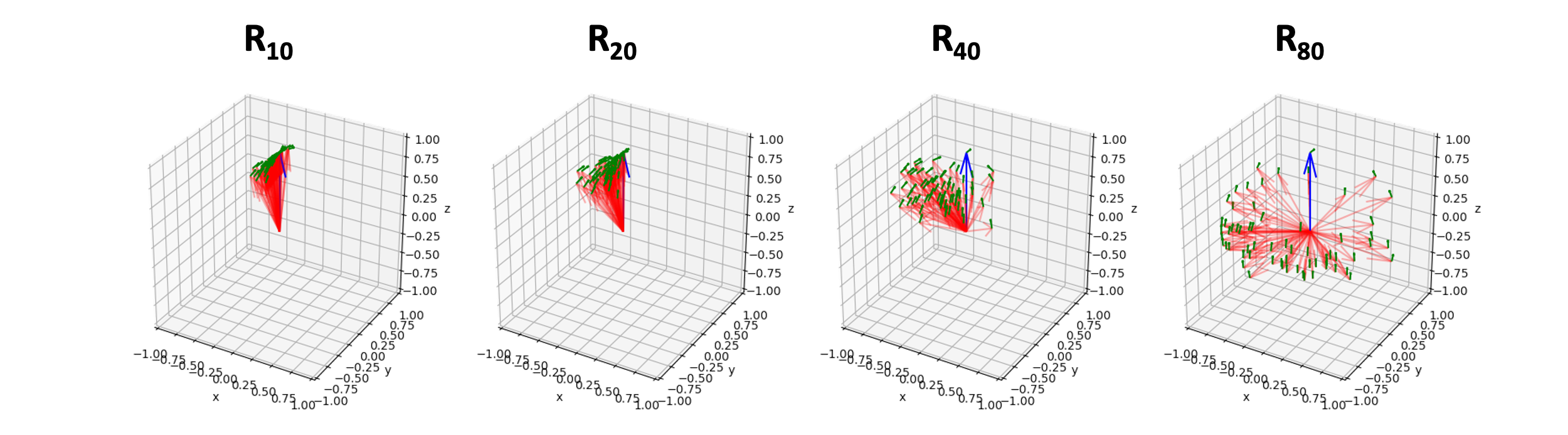


Figure S1 An illustration of the randomly sampled rotations with a mean pitch angle of 10⁰, 20⁰, 40⁰ and 80⁰, and standard deviation of 10⁰. The blue arrows represent the original vector, and the red arrows represent the rotated vector, the small green arrows are extra orientation references attached to the tip of the blue or red arrows. In each plot, a total of 64 rotations were sampled and visualized. These four sets of rotation represent different degrees of synthetic deviation in orientation from the original scan applied about the patients’ tumours in the dataset.

## Details of radiomics model building pipeline

### Brief summary

In this study, we trained radiomics models with texture features derived from unrotated WD and nWD filtered-images. These models were then evaluated using corresponding features obtained from images that had been rotated. This setup replicates the clinical scenario in which tumours in the new patients present orientation variations that are not aligned with those in the patient dataset used for training the models. This design aims to enhance the clinical utility of radiomics by evaluating the effect of inherent variability in tumor growth orientation that is difficult, if not impossible, to account for during imaging and model training.

### Phase i) — Preliminary feature selection

Features selection was implemented to reduce the data dimensionality and mitigate risks of overfitting. In this study, there are two levels of features selection – preliminary feature selection and fine feature selection. Preliminary feature selection was performed prior to K-fold cross-validation based on general statistical properties of the features. Subsequent fine feature selection was carried out exclusively on the training data during the repeated K-fold cross-validation step, covered in the next section, utilizing the supervised elastic net technique. This dual-stage feature selection strategy strikes a balance between computational efficiency and the minimization of data leakage between training and testing sets, thereby ensuring the robustness of our results.

In the preliminary feature selection phase, the 1674 texture features were analysed statistically to identify features significantly associate with NSCLC subtypes. First, a variance threshold filter was used to remove features with a very small variance < 1 × 10^-4^, indicating near-uniform values across all samples—often products of erroneous numerical computation during image filtering or texture calculations. Second, ANOVA or Mann-Whitney U-Test, depending on the feature normality test results, was used to identify features that have significantly different group mean across the three NSCLC subtypes groups. Radiomic features that failed to meet the significance threshold of p-values < 0.05 were excluded.

### Phase ii) — Supervised fine feature selection

For the supervised fine feature selection, we adopted the elastic net regularization technique, with the regularization parameter (alpha) empirically configured at 0.01 and the L1 ratio at 0.9. The elastic net can be viewed as a hybrid of L1 and L2 regularization, as seen in LASSO and Ridge regression techniques, respectively, with the L1 ratio dictating the balance between the two. During the repeated K-fold cross-validation, detailed in the following section, the elastic net was applied to the training folds to further select features with the best potential to discriminate between NSCLC subtypes. Given the sensitivity of elastic net to class imbalance owing to the L1 regularization component, we incorporated the Synthetic Minority Oversampling Technique (SMOTE) to synthesize minority ADC samples in this study. It is critical to note that SMOTE was not applied to the testing samples to inflate the estimation the model performance. Features assigned a zero coefficient by the elastic net were deemed non-contributory and thus excluded from subsequent model building step.

A comprehensive flow-chart to explain the feature selection pipeline is given in Figure S2.


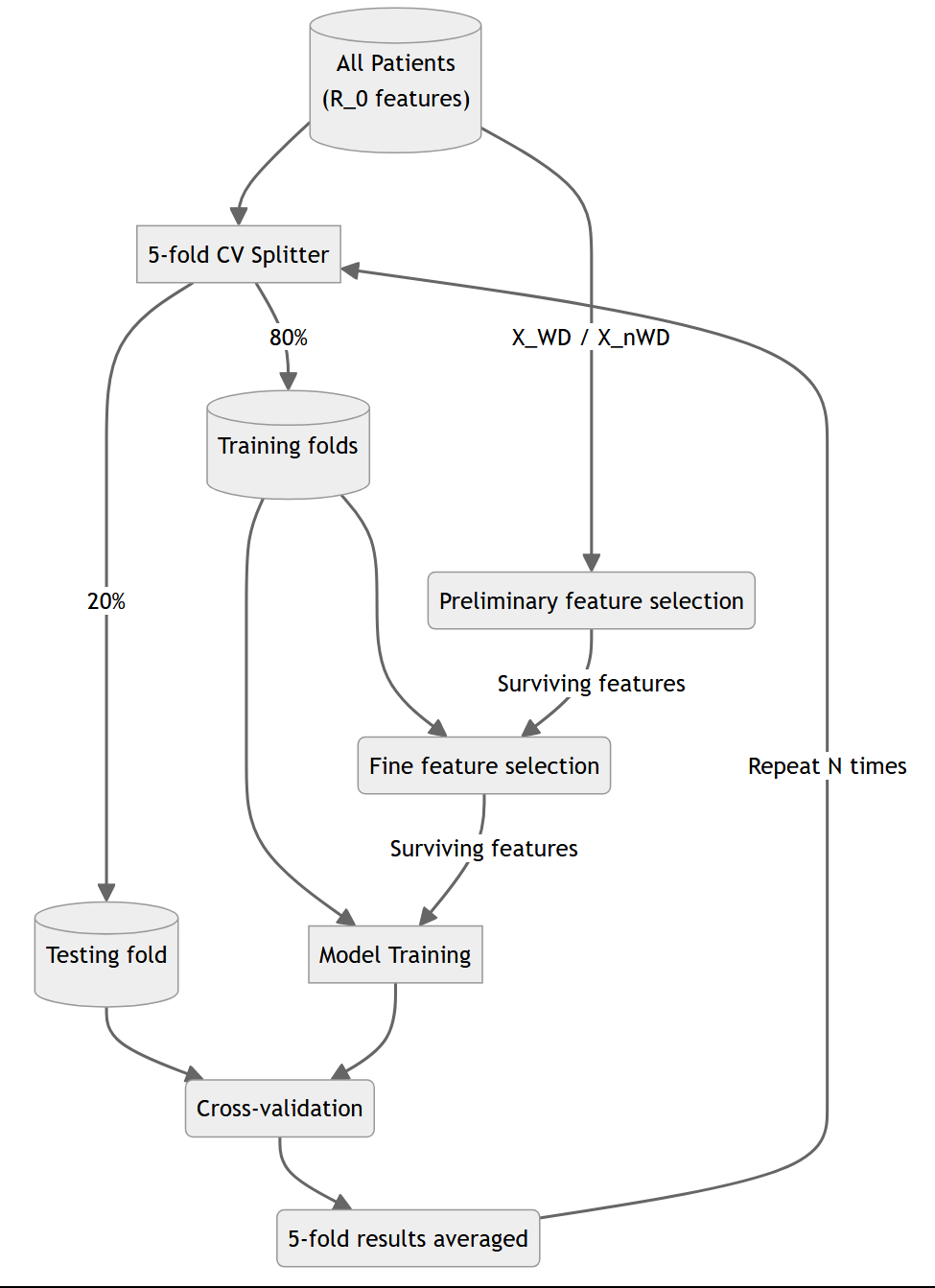


Figure S2 A flow-chart describing the feature selection pipeline and how it interacts with the performance validation analysis, which is conducted by repeating 5-fold cross-validation many times. CV = cross-validation, X_WD = wavelet decomposition features, X_nWD = non-WD features.

### Phase iii) — Model building

In the model building phase, various classifiers were trained with their hyperparameters optimized using the grid search strategy, detailed below:

#### Classifiers

In this study, we tested the performance of 5 classifiers to discriminate between three NSCLC subtypes ADC, SCC and LCC. The 5 classifiers are K-nearest neighbours (KNN), logistic regression (LR), random forest (RF), support vector machine (SVM) with linear kernel, and SVM with RBF kernels (SVM_rbf_). We adopted the implementations of these classifiers provided by the open-source Python package “sklearn” version v1.0.1 [5]. These five classifiers are well-established in the literature and were chosen as they inherently allow for multi-class classification that was necessary in this study such that no additional modification was required.

To improve generalizability of the models, all classifiers are paired with a feature value normalizer. We utilized the “StandardScaler” offered by the “sklearn” python package [5] in this study, which scales and shift each feature to achieve unit variance and zero mean across samples.

$$x^{'}=\frac{x-Mean(X)}{SD(X)}$$

#### Grid search for optimal training hyperparameters

During each run of the repeated 5-fold cross-validation, we performed a grid-search over the training folds to determine the optimal hyperparameters combination for each classifier. It was conducted through the “GridSearchCV” function provided by the “sklearn” (v1.0.1) package. This function took a pipeline of steps that are sequentially executed, comprising the feature normalization step (“standardization”) and the classification step, and the hyperparameter search grid as input. It iterates all possible combinations of the search-grid to identify the combination of hyperparameters with the best performance based on 5-fold cross-validation. After all possible combinations are tested, the one with the best performance was selected as the optimal hyperparameters and saved for later. The hyperparameter search grid used in this study was empirically implemented and reported below.

##### KNN

- Number of neighbours: [3, 5, 7, 10, 20, 30]

##### RF

- Number of trees: [20, 30, 50, 100]
- Loss function: [Gini impurity, Shannon’s entropy]
- Maximum node depth: [No restriction, 5, 10, 20]

##### LR

- Elastic net regularization penalty ($1/C$): [10, 1, 0.01, 0.001]
- Elastic net L_1_ ratio: [0.1, 0.9]

##### SVM

- Regularization parameter: [1, 10, 100]

##### SVM_rbf_

- Regularization parameter: [1, 10, 100]
- Kernel coefficient ($\gamma$): [1 / N_features_, 1 / [N_features_ × VAR(x)]]
- Use shrinking: [True, False]

During the grid search, we used NSCLC subtype weighted prediction accuracy as the performance metrics to determine the optimal hyperparameters. The weighted accuracy accounts for the class imbalance between the three tested NSCLC subtypes by weighting “hits” of different classes. For each classifier and its hyperparameter combination, 5-fold cross-validation was conducted and the performance was determined by averaging the performance across the cross-validation. The hyperparameter combination with the best average performance was recorded to be used later.

#### Models training and performance validation

To systematically assess the impact of orientation deviation on classifier performance, classifiers were trained using the optimal hyperparameters identified in the previous grid-search step, using only features from the original orientation ($R_{0}$). These classifiers were tested for their robustness against features extracted from testing set scans including their rotated counterpart ($R_{0}$ to $R_{80}$). To ensure the reliability of our findings and to account for potential sampling bias, we repeated the 5-fold cross-validation and observe if the results fluctuate.

Specifically, we repeated the 5-fold cross-validation step for 50 times. In each iteration, the dataset was randomly partitioned into 5 folds with stratification applied to maintain a consistent class distribution of NSCLC subtypes across folds. This stratification ensured that each fold contained a proportional representation of the subtypes, mirroring that of the entire dataset. Within each fold of the cross-validation, 5 classifiers were trained to discriminate between NSCLC subtypes, employing a one-versus-rest (OVR) strategy. This strategy trains three separate instances of the same classifier, each tasked with binary classification of one of the three NSCLC subtypes against the remaining two. These individual classifiers were then aggregated to create a multi-class ensemble classification model.

For validation of performance, the radiomics models that were trained using $R_{0}$ features in the training fold were validated using $R_{0}$ to $R_{80}$ features of the testing fold. Given that rotated features exhibit distinct means and standard deviations, as described on the feature-level analysis of this study, we fine-tunned the feature-normalization parameters. These adjustments were derived from the training set $R_{05}$ to $R_{80}$ features to avoid data leakage between training and testing sets. It is important to note that the classifiers that were initially trained on $R_{0}$ features underwent no retraining or fine-tunning.

#### Validation metric

The validation metric used in this study is the conventional accuracy, calculated from the number of corrected prediction over all predictions:

$$ACC=\frac{1}{N}\sum_{n=1}^{N} I(x_{n}, y_{n})$$

Where $x_{n}$ and $y_{n}$ are the predictions and ground-truth histology for the $n$-th sample, respectively, and $I(a, b)$ is the one-hot encoder function that is 1 when $a=b$ and 0 otherwise:

$$I\left( a, b \right)=\left\{ \begin{matrix} 1 & \mathrm{if}a=b \\ 0 & \mathrm{otherwise} \end{matrix} \right.$$

Of all tested classifiers, only LR produces multiple, one for each class, continuous values inherently as its predictions. In this case, the class with the largest prediction value is taken as its final prediction $x_{n}$.

# Results

## Detail rationales of patient exclusion

Prior to phase 1 of the study (feature-level analysis), we inspected all NSCLC patients’ images and segmentation released by Aerts et al. [6] and found two patients (LUNG1-128 and LUNG1-246) with a mismatched spatial information between their CT scans and manual segmentation, and one patient with an suspected erroneous segmentation (LUNG1-019). These 3 patients were excluded from all analysis. A screenshot of the segmentation visualized overlaying the CT scan patient LUNG1-019 is given below:


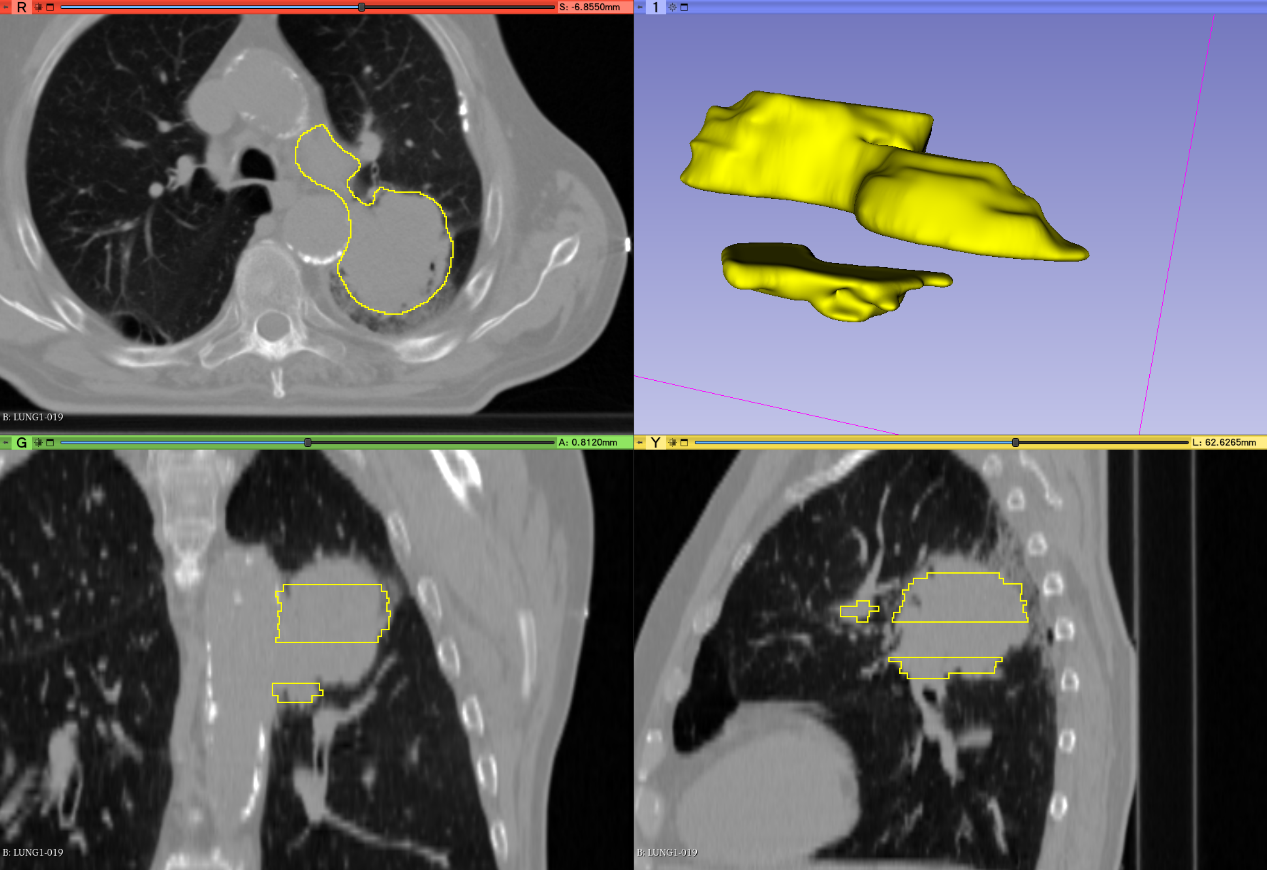


Figure S3 Screenshot of CT scan of a NSCLC patient (LUNG1-019) and the manually drawn contour (yellow) of the neoplasm provided by Aerts et al. [6]. The image shows potential erroneous segmentation which lead to the exclusion of this case from the current study.

Prior to phase 2 (performance level analysis), we further excluded 3 more patients with individual GTV segmentations on both sides of the lung (LUNG1-326, LUNG1-372, LUNG1-399) in addition to the exclusion of patients labelled as “nos” or “N/A”. There was a lack of definitive information to map the reported histology a specific segmentation on the CT scans. Since the purpose of phase 2 was to evaluate radiomic model performance to discriminate between NSCLC subtypes, we excluded these patients. Note that these patients were not excluded in the first phase because the presence of histological information does not challenge the hypothesis that radiomic feature values from the same lesion should remain consistent irrespective of the imaging orientation.

## Performance of model evaluate in more metrics


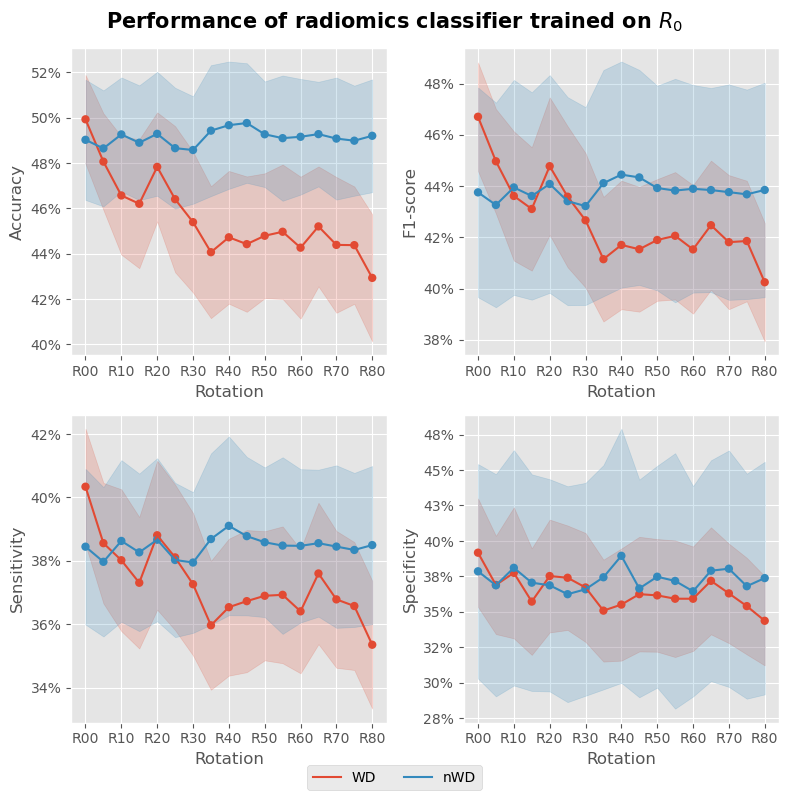


Figure S4 Summary plot of radiomics model performance built from WD and non-WD features to discriminate between three major subtypes of non-small-cell lung cancer, namely large cell carcinoma, squamous cell carcinoma and adenocarcinoma. The models were trained on training-fold $R_{0}$ features (i.e., original orientation), and then tested on testing-fold $R_{0}$ to $R_{80}$ features. The f1-score, sensitivity and specificity were evaluated in one-versus-rest manner.

# Supplementary References

1 Wong LM (2023) MRI Normalization Tools. GitHub. Available via https://github.com/alabamagan/mri_normalization_tools/tree/main. Accessed Jan 18th 2023

2 van Griethuysen JJM, Fedorov A, Parmar C et al (2017) Computational Radiomics System to Decode the Radiographic Phenotype. Cancer Res 77:e104-e107

3 van Griethuysen JJM, Fedorov A, Parmar C et al (2022) Documentation - pyradiomics v3.0. Available via https://pyradiomics.readthedocs.io/en/v3.0/. Accessed Dec 20th 2023

4 Banerjee J, Moelker A, Niessen WJ, van Walsum T (2013) 3D LBP-based rotationally invariant region descriptionComputer Vision-ACCV 2012 Workshops: ACCV 2012 International Workshops, Daejeon, Korea, November 5-6, 2012, Revised Selected Papers, Part I 11. Springer, pp 26-37

5 Pedregosa F, Varoquaux G, Gramfort A et al (2011) Scikit-learn: Machine Learning in Python. Journal of Machine Learning Research 12:2825-2830

6 Aerts HJWL, Wee L, Rios Velazquez E et al, Data From NSCLC-Radiomics (version 4) (2014). [Dataset] Accesible via https://doi.org/10.7937/K9/TCIA.2015.PF0M9REI Accessed 13 Sept 2023
