# Supplementary material for: Decoding the Rotation Effect: A Retrospective Analysis of Lesion Orientation and Its Impact on Wavelet-Based Radiomics Feature Extraction and Lung Cancer Classification
Source: J Imaging Inform Med. 2025 May 6;39(1):265–76. doi: 10.1007/s10278-025-01520-8 (PMC12920822; doi:10.1007/s10278-025-01520-8)
Supplement: Supplementary file 2 — Supplementary file2 (DOCX 48 KB) [file 10278_2025_1520_MOESM2_ESM.docx]

Supplemental material – Appendix B: Tables

# Results

## Feature-level analysis results

Table S1 Results of Spearman’s rank test between shape feature values and applied degree of rotation. Ideal radiomic features should show no correlation as it should remain a constant regardless of scan orientation. Bold font indicates statistical significance (p < .05).

| **Feature Name** | **Spearman’s CC** | **p-value** |
| --- | --- | --- |
| *Elongation* | 0.000 | 0.999 |
| *Flatness* | 0.000 | 0.978 |
| *Least Axis Length* | -0.001 | 0.940 |
| *Major Axis Length* | -0.001 | 0.947 |
| *Maximum2D Diameter Column* | -0.025 | **0.028** |
| *Maximum2D Diameter Row* | -0.008 | 0.484 |
| *Maximum2D Diameter Slice* | 0.024 | **0.033** |
| *Maximum3D Diameter* | -0.001 | 0.944 |
| *Mesh Volume* | -0.001 | 0.941 |
| *Minor Axis Length* | -0.001 | 0.949 |
| *Sphericity* | -0.020 | 0.076 |
| *Surface Area* | 0.001 | 0.898 |
| *Surface Volume Ratio* | 0.004 | 0.705 |
| *Voxel Volume* | -0.001 | 0.958 |

CC = Spearman’s correlation coefficient, 2D = 2-dimensional

Table S2 Summary of inter-quartile rage (IQR) percentage differences ($\boldsymbol{\%\Delta}$) of feature values before and after applying rotations grouped by imaging filter. The IQR of $\boldsymbol{\%\Delta}$ reflects the value instability against rotations. The percentage of features with Spearman’s rank test significance suggest the portion of texture that showed significant trend associated with the degree of the rotation applied.

| Imaging Filter | IQR of $\boldsymbol{\%}\boldsymbol{\Delta}$  Mean (95% CI) | % features filtered  by variance | % features with  sig. trend^a^ |
| --- | --- | --- | --- |
| **WD (n=744)** |  |  |  |
| ***Combined*** | 34.0% (32.7%-35.4%) | 1.61% (12/744) | 23.66% (176/744) |
| *wavelet-HHH* | 35.8% (32.5%-39.0%) | 9.68% (9/93) | 13.98% (13/93) |
| *wavelet-HHL* | 42.0% (38.6%-45.3%) | 1.08% (1/93) | 33.33% (31/93) |
| *wavelet-HLH* | 36.1% (32.7%-39.5%) | 1.08% (1/93) | 36.56% (34/93) |
| *wavelet-HLL* | 14.4% (13.6%-15.3%) | 0.00% (0/93) | 0.00% (0/93) |
| *wavelet-LHH* | 35.7% (32.9%-38.5%) | 1.08% (1/93) | 16.13% (15/93) |
| *wavelet-LHL* | 48.1% (41.3%-54.9%) | 0.00% (0/93) | 35.48% (33/93) |
| *wavelet-LLH* | 56.9% (52.7%-61.2%) | 0.00% (0/93) | 53.76% (50/93) |
| *wavelet-LLL* | 3.2% (3.0%-3.4%) | 0.00% (0/93) | 0.00% (0/93) |
| **nWD (n=930)** |  |  |  |
| ***Combined*** | 7.3% (6.6%-7.9%) | 0.00% (0/930) | 0.54% (5/930) |
| *Exponential* | 6.4% (5.8%-7.0%) | 0.00% (0/93) | 0.00% (0/93) |
| *Gradient* | 6.5% (5.9%-7.1%) | 0.00% (0/93) | 0.00% (0/93) |
| *LBP_3D-k_* | 4.7% (4.4%-5.0%) | 0.00% (0/93) | 0.00% (0/93) |
| *LBP_3D-m1_* | 3.0% (2.7%-3.2%) | 0.00% (0/93) | 0.00% (0/93) |
| *LBP_3D-m2_* | 6.8% (6.4%-7.3%) | 0.00% (0/93) | 4.30% (4/93) |
| *LoG* | 4.4% (4.0%-4.8%) | 0.00% (0/93) | 0.00% (0/93) |
| *Logarithm* | 7.0% (6.7%-7.3%) | 0.00% (0/93) | 0.00% (0/93) |
| *Original* | 4.9% (4.6%-5.3%) | 0.00% (0/93) | 1.08% (1/93) |
| *Square* | 23.1% (16.8%-29.4%) | 0.00% (0/93) | 0.00% (0/93) |
| *Square root* | 6.1% (5.8%-6.3%) | 0.00% (0/93) | 0.00% (0/93) |

^a^ Significant CC is defined as Spearman's rank test p-value < .05 and magnitude of correlation coefficient ≥ 0.1; WD = Wavelet decomposition, nWD = non-WD, IQR = Interquartile range, LBP = local binary pattern, LoG = Laplacian of Gaussian, CC = Spearman’s correlation coefficient.

Table S3 Summary of inter-quartile rage (IQR) of percentage differences ($\boldsymbol{\%}\boldsymbol{\Delta}$) of feature values before and after applying rotations grouped by texture category. Overall, wavelet (WD) features showed higher IQR of $\boldsymbol{\%}\boldsymbol{\Delta}$ compared to non-WD (nWD) features. More WD features (23.7%) displayed significant association with rotated degrees when compared to nWD features (0.5%). Among texture categories, the IQR of $\boldsymbol{\%}\boldsymbol{\Delta}$ were greatest for first order and gray-level size zone matrix (GLSZM) textures for both WD and nWD textures. GLCM = gray-level co-occurrence matrix, GLDM = gray-level dependence matrix, GLRLM = gray-level run length matrix, GLSZM = gray-level size zone matrix, NGTDM = neighborhood gray tone difference matrix.

| Texture Category | IQR of $\boldsymbol{\%}\boldsymbol{\Delta}$  Mean (95% CI) | % features filtered  by variance | % features with sig. trend^a^ |
| --- | --- | --- | --- |
| **WD filters (n=744)** |  |  |  |
| **Combined** | 34.0% (32.7%-35.4%) | 1.61% (12/744) | 23.66% (176/744) |
| Firstorder | 56.5% (53.3%-59.8%) | 3.47% (5/144) | 31.25% (45/144) |
| GLCM | 15.1% (13.9%-16.3%) | 0.52% (1/192) | 27.08% (52/192) |
| GLDM | 18.4% (16.3%-20.5%) | 0.89% (1/112) | 19.64% (22/112) |
| GLRLM | 16.3% (14.5%-18.0%) | 3.91% (5/128) | 20.31% (26/128) |
| GLSZM | 64.7% (59.8%-69.5%) | 0.00% (0/128) | 17.97% (23/128) |
| NGTDM | 46.1% (37.2%-55.1%) | 0.00% (0/40) | 20.00% (8/40) |
| **nWD (n = 930)** |  |  |  |
| **Combined** | 7.3% (6.6%-7.9%) | 0.00% (0/930) | 0.54% (5/930) |
| Firstorder | 10.3% (7.1%-13.6%) | 0.00% (0/180) | 1.11% (2/180) |
| GLCM | 3.4% (3.2%-3.5%) | 0.00% (0/240) | 0.00% (0/240) |
| GLDM | 4.1% (3.9%-4.3%) | 0.00% (0/140) | 0.00% (0/140) |
| GLRLM | 3.2% (3.1%-3.3%) | 0.00% (0/160) | 0.62% (1/160) |
| GLSZM | 16.5% (16.0%-17.0%) | 0.00% (0/160) | 1.25% (2/160) |
| NGTDM | 7.8% (7.2%-8.4%) | 0.00% (0/50) | 0.00% (0/50) |

^a^ Significant CC is defined as Spearman's rank test p-value < .05 and magnitude of correlation coefficient ≥ 0.3.

## Performance-level analysis

**Table S4** Performance of models grouped by classifier averaged across 50 repetitions of 5-fold cross-validation in accuracy. The models were trained on training-fold $R_{0}$ features (i.e., original orientation), and then tested on testing-fold $R_{0}$ to $R_{80}$ features. Data displayed is mean ± sd.

| **Rotations** | **KNN** | **Logistic Regression** | **Random Forest** | **Support Vector Machine** | **Support Vector Machine (rbf)** | **All** |
| --- | --- | --- | --- | --- | --- | --- |
| **WD** |  |  |  |  |  |  |
| **R00** | 48.6% ± 1.8% | 50.0% ± 1.8% | 50.3% ± 1.9% | 50.4% ± 1.7% | 50.4% ± 2.1% | 49.9% ± 2.0% |
| **R05** | 47.3% ± 2.0% | 47.1% ± 2.1% | 48.9% ± 2.4% | 48.4% ± 1.8% | 48.5% ± 1.7% | 48.1% ± 2.1% |
| **R10** | 44.5% ± 2.0% | 47.3% ± 2.4% | 47.7% ± 2.1% | 48.0% ± 2.3% | 45.3% ± 2.4% | 46.6% ± 2.6% |
| **R15** | 44.5% ± 2.0% | 44.8% ± 2.8% | 49.2% ± 1.8% | 46.2% ± 2.6% | 46.3% ± 2.3% | 46.2% ± 2.8% |
| **R20** | 45.9% ± 1.9% | 48.9% ± 2.1% | 47.0% ± 1.8% | 49.1% ± 2.6% | 48.2% ± 2.0% | 47.8% ± 2.4% |
| **R25** | 42.7% ± 1.7% | 46.3% ± 2.9% | 49.5% ± 2.2% | 47.1% ± 2.7% | 46.4% ± 2.2% | 46.4% ± 3.2% |
| **R30** | 43.8% ± 2.1% | 43.7% ± 3.5% | 48.3% ± 1.7% | 45.0% ± 3.3% | 46.1% ± 2.1% | 45.4% ± 3.1% |
| **R35** | 41.8% ± 2.2% | 43.1% ± 2.4% | 47.0% ± 2.2% | 44.2% ± 3.1% | 44.3% ± 1.8% | 44.1% ± 2.9% |
| **R40** | 42.5% ± 2.2% | 43.3% ± 2.7% | 47.0% ± 2.1% | 45.1% ± 3.1% | 45.7% ± 2.0% | 44.7% ± 2.9% |
| **R45** | 46.3% ± 2.3% | 41.6% ± 2.2% | 46.8% ± 1.9% | 42.7% ± 2.7% | 44.7% ± 2.0% | 44.4% ± 3.0% |
| **R50** | 44.0% ± 2.1% | 43.0% ± 2.7% | 47.2% ± 2.1% | 44.8% ± 2.8% | 45.0% ± 2.1% | 44.8% ± 2.8% |
| **R55** | 43.5% ± 2.1% | 43.2% ± 2.6% | 47.6% ± 2.3% | 44.9% ± 3.2% | 45.6% ± 2.2% | 45.0% ± 3.0% |
| **R60** | 43.7% ± 1.9% | 41.9% ± 2.4% | 47.8% ± 1.8% | 43.5% ± 3.3% | 44.5% ± 2.5% | 44.3% ± 3.1% |
| **R65** | 43.1% ± 2.0% | 44.2% ± 2.2% | 46.0% ± 2.4% | 45.3% ± 2.4% | 47.4% ± 1.7% | 45.2% ± 2.6% |
| **R70** | 42.0% ± 2.3% | 43.2% ± 2.4% | 47.8% ± 2.1% | 44.5% ± 2.7% | 44.5% ± 2.1% | 44.4% ± 3.0% |
| **R75** | 42.7% ± 2.3% | 43.1% ± 2.3% | 46.3% ± 2.0% | 44.7% ± 2.9% | 45.1% ± 1.5% | 44.4% ± 2.6% |
| **R80** | 41.8% ± 2.1% | 40.7% ± 2.5% | 44.9% ± 2.3% | 43.1% ± 2.6% | 44.2% ± 2.4% | 42.9% ± 2.8% |
| **nWD** |  |  |  |  |  |  |
| **R00** | 45.9% ± 1.9% | 51.0% ± 1.7% | 49.1% ± 1.9% | 50.9% ± 1.8% | 48.3% ± 2.0% | 49.0% ± 2.7% |
| **R05** | 45.6% ± 2.0% | 50.6% ± 1.7% | 48.7% ± 2.0% | 50.1% ± 1.4% | 48.1% ± 2.1% | 48.6% ± 2.6% |
| **R10** | 46.5% ± 1.7% | 51.5% ± 1.6% | 49.0% ± 1.9% | 51.0% ± 1.3% | 48.3% ± 2.1% | 49.3% ± 2.5% |
| **R15** | 46.0% ± 2.0% | 51.2% ± 1.5% | 48.4% ± 2.1% | 50.6% ± 1.2% | 48.2% ± 1.7% | 48.9% ± 2.5% |
| **R20** | 46.0% ± 2.4% | 51.5% ± 1.6% | 49.5% ± 1.9% | 51.1% ± 1.4% | 48.4% ± 2.0% | 49.3% ± 2.7% |
| **R25** | 45.2% ± 2.0% | 50.5% ± 1.7% | 48.8% ± 1.9% | 50.6% ± 1.2% | 48.1% ± 2.0% | 48.7% ± 2.7% |
| **R30** | 45.9% ± 2.1% | 50.2% ± 1.7% | 48.7% ± 1.9% | 50.0% ± 1.4% | 48.1% ± 1.9% | 48.6% ± 2.4% |
| **R35** | 46.3% ± 2.7% | 51.8% ± 1.7% | 49.2% ± 1.9% | 51.7% ± 1.4% | 48.2% ± 2.0% | 49.4% ± 2.9% |
| **R40** | 46.4% ± 1.9% | 52.5% ± 1.7% | 49.2% ± 2.2% | 51.5% ± 1.4% | 48.7% ± 1.8% | 49.7% ± 2.8% |
| **R45** | 47.0% ± 2.1% | 51.5% ± 1.8% | 49.2% ± 2.0% | 52.2% ± 1.4% | 48.8% ± 2.1% | 49.8% ± 2.6% |
| **R50** | 47.2% ± 1.8% | 51.0% ± 1.6% | 48.5% ± 2.0% | 51.0% ± 1.4% | 48.6% ± 2.0% | 49.3% ± 2.3% |
| **R55** | 45.6% ± 1.8% | 51.8% ± 1.4% | 48.7% ± 1.7% | 51.2% ± 1.4% | 48.1% ± 1.8% | 49.1% ± 2.8% |
| **R60** | 46.2% ± 2.0% | 51.4% ± 1.7% | 49.1% ± 2.0% | 50.5% ± 1.5% | 48.6% ± 2.0% | 49.2% ± 2.5% |
| **R65** | 47.2% ± 2.2% | 51.0% ± 1.5% | 48.9% ± 2.0% | 50.7% ± 1.3% | 48.5% ± 2.0% | 49.3% ± 2.3% |
| **R70** | 45.9% ± 2.0% | 51.6% ± 1.6% | 49.0% ± 2.1% | 50.8% ± 1.4% | 48.1% ± 1.9% | 49.1% ± 2.7% |
| **R75** | 46.5% ± 2.0% | 51.2% ± 1.5% | 48.6% ± 2.0% | 50.5% ± 1.5% | 48.2% ± 1.6% | 49.0% ± 2.4% |
| **R80** | 46.8% ± 1.8% | 51.4% ± 1.6% | 48.7% ± 1.8% | 51.0% ± 1.5% | 48.0% ± 2.1% | 49.2% ± 2.5% |

WD = wavelet decomposition, nWD = non-WD, KNN = K-nearest neighbours, rbf = radial basis function

**Table S5** The list of all features selected during for wavelet-feature (WD)-only training during the 50 repetitions of 5-fold cross-validation, their counts, and the probability of being selected. Out of 744 WD features, the 32 features tabulated here are consistently selected despite the training sample differs in each run, indicating the robustness of feature selection scheme in our pipeline.

| **Selected feature [Imaging filter, texture category, feature name]** | **Counts** | **Probability** |
| --- | --- | --- |
| (wavelet-LLH, glcm, Cluster Tendency) | 250 | 100.00% |
| (wavelet-HHL, glcm, Cluster Tendency) | 250 | 100.00% |
| (wavelet-LHL, glszm, Low Gray Level Zone Emphasis) | 250 | 100.00% |
| (wavelet-LHH, glrlm, Run Variance) | 249 | 99.60% |
| (wavelet-HLH, glcm, MCC) | 249 | 99.60% |
| (wavelet-LLH, glszm, Small Area Low Gray Level Emphasis) | 248 | 99.20% |
| (wavelet-HHH, glrlm, Short Run Low Gray Level Emphasis) | 247 | 98.80% |
| (wavelet-LLH, glrlm, Short Run Emphasis) | 247 | 98.80% |
| (wavelet-LHH, firstorder, Interquartile Range) | 247 | 98.80% |
| (wavelet-LLH, firstorder, Minimum) | 242 | 96.80% |
| (wavelet-HLL, gldm, Large Dependence Emphasis) | 239 | 95.60% |
| (wavelet-HLL, firstorder, Interquartile Range) | 231 | 92.40% |
| (wavelet-HHL, glcm, Contrast) | 221 | 88.40% |
| (wavelet-LHH, ngtdm, Contrast) | 216 | 86.40% |
| (wavelet-LHL, gldm, Large Dependence Emphasis) | 212 | 84.80% |
| (wavelet-LLH, glrlm, Short Run High Gray Level Emphasis) | 211 | 84.40% |
| (wavelet-HHL, glcm, Cluster Prominence) | 199 | 79.60% |
| (wavelet-HLH, glszm, Gray Level Variance) | 199 | 79.60% |
| (wavelet-HHL, firstorder, Variance) | 196 | 78.40% |
| (wavelet-LHH, glcm, Idn) | 182 | 72.80% |
| (wavelet-HLH, firstorder, Interquartile Range) | 177 | 70.80% |
| (wavelet-HLH, glrlm, Low Gray Level Run Emphasis) | 172 | 68.80% |
| (wavelet-HLH, glrlm, Long Run High Gray Level Emphasis) | 156 | 62.40% |
| (wavelet-HHL, firstorder, 90Percentile) | 144 | 57.60% |
| (wavelet-HHH, gldm, Large Dependence High Gray Level Emphasis) | 144 | 57.60% |
| (wavelet-LHH, glrlm, Short Run Low Gray Level Emphasis) | 142 | 56.80% |
| (wavelet-LHH, gldm, Low Gray Level Emphasis) | 141 | 56.40% |
| (wavelet-HLH, ngtdm, Contrast) | 134 | 53.60% |
| (wavelet-LHH, glrlm, Short Run High Gray Level Emphasis) | 130 | 52.00% |
| (wavelet-LLH, glrlm, High Gray Level Run Emphasis) | 129 | 51.60% |
| (wavelet-HHL, firstorder, 10Percentile) | 101 | 40.40% |
| (wavelet-HLH, ngtdm, Complexity) | 60 | 24.00% |

L = low-pass filter, H = high-pass filter, glcm = gray-level co-occurrence matrix, glszm = gray-level size-zone matrix, glrlm = gray-level run-length matrix, gldm = gray-level dependence, ngtdm = neighbouring gray tone difference matrix, MCC = maximal correlation coefficient, ldn = inverse difference normalized.

**Table S6** The list of all features selected during for non-wavelet-feature (nWD)-only training during the 50 repetitions of 5-fold cross-validation, their counts, and the probability of being selected. Out of 930 nWD features, the 26 features tabulated here are consistently selected despite the training sample differs in each run, indicating the robustness of feature selection scheme in our pipeline.

| **Selected feature [Imaging filter, texture category, feature name]** | **Counts** | **Probability** |
| --- | --- | --- |
| (lbp-3D-k, glszm, Zone Percentage) | 250 | 100.00% |
| (exponential, glszm, Size Zone Non-Uniformity Normalized) | 250 | 100.00% |
| (lbp-3D-m1, glcm, Correlation) | 250 | 100.00% |
| (exponential, firstorder, Kurtosis) | 247 | 98.80% |
| (exponential, ngtdm, Strength) | 247 | 98.80% |
| (squareroot, gldm, Dependence Non-Uniformity Normalized) | 246 | 98.40% |
| (exponential, glcm, Cluster Shade) | 246 | 98.40% |
| (square, firstorder, 10Percentile) | 246 | 98.40% |
| (logarithm, glcm, Inverse Variance) | 246 | 98.40% |
| (lbp-3D-k, glszm, Small Area Low Gray Level Emphasis) | 242 | 96.80% |
| (lbp-3D-m1, glszm, Small Area Low Gray Level Emphasis) | 236 | 94.40% |
| (squareroot, firstorder, Uniformity) | 232 | 92.80% |
| (squareroot, glcm, Maximum Probability) | 229 | 91.60% |
| (exponential, glcm, Cluster Prominence) | 218 | 87.20% |
| (logarithm, gldm, Dependence Non-Uniformity Normalized) | 215 | 86.00% |
| (squareroot, glrlm, Run Entropy) | 212 | 84.80% |
| (logarithm, glcm, Maximum Probability) | 199 | 79.60% |
| (square, glszm, Gray Level Variance) | 195 | 78.00% |
| (exponential, firstorder, Skewness) | 184 | 73.60% |
| (lbp-3D-k, glszm, Zone Entropy) | 172 | 68.80% |
| (lbp-3D-m1, glszm, Low Gray Level Zone Emphasis) | 167 | 66.80% |
| (lbp-3D-k, glszm, Gray Level Non-Uniformity Normalized) | 159 | 63.60% |
| (lbp-3D-k, gldm, High Gray Level Emphasis) | 156 | 62.40% |
| (squareroot, glrlm, Run Percentage) | 99 | 39.60% |
| (squareroot, glcm, Idm) | 87 | 34.80% |
| (logarithm, glcm, Idn) | 50 | 20.00% |

lbp = local binary pattern, 3D = 3-dimensional, lglcm = gray-level co-occurrence matrix, glszm = gray-level size-zone matrix, glrlm = gray-level run-length matrix, gldm = gray-level dependence, ngtdm = neighbouring gray tone difference matrix, Idm = inverse difference moment, ldn = inverse difference normalized.
