# Supplementary material for: Decoding the Rotation Effect: A Retrospective Analysis of Lesion Orientation and Its Impact on Wavelet-Based Radiomics Feature Extraction and Lung Cancer Classification
Source: J Imaging Inform Med. 2025 May 6;39(1):265–76. doi: 10.1007/s10278-025-01520-8 (PMC12920822; doi:10.1007/s10278-025-01520-8)
Supplement: Supplementary file 3 — Supplementary file3 (DOCX 12278 KB) [file 10278_2025_1520_MOESM3_ESM.docx]

Supplementary material - Appendix C: Additional Figures


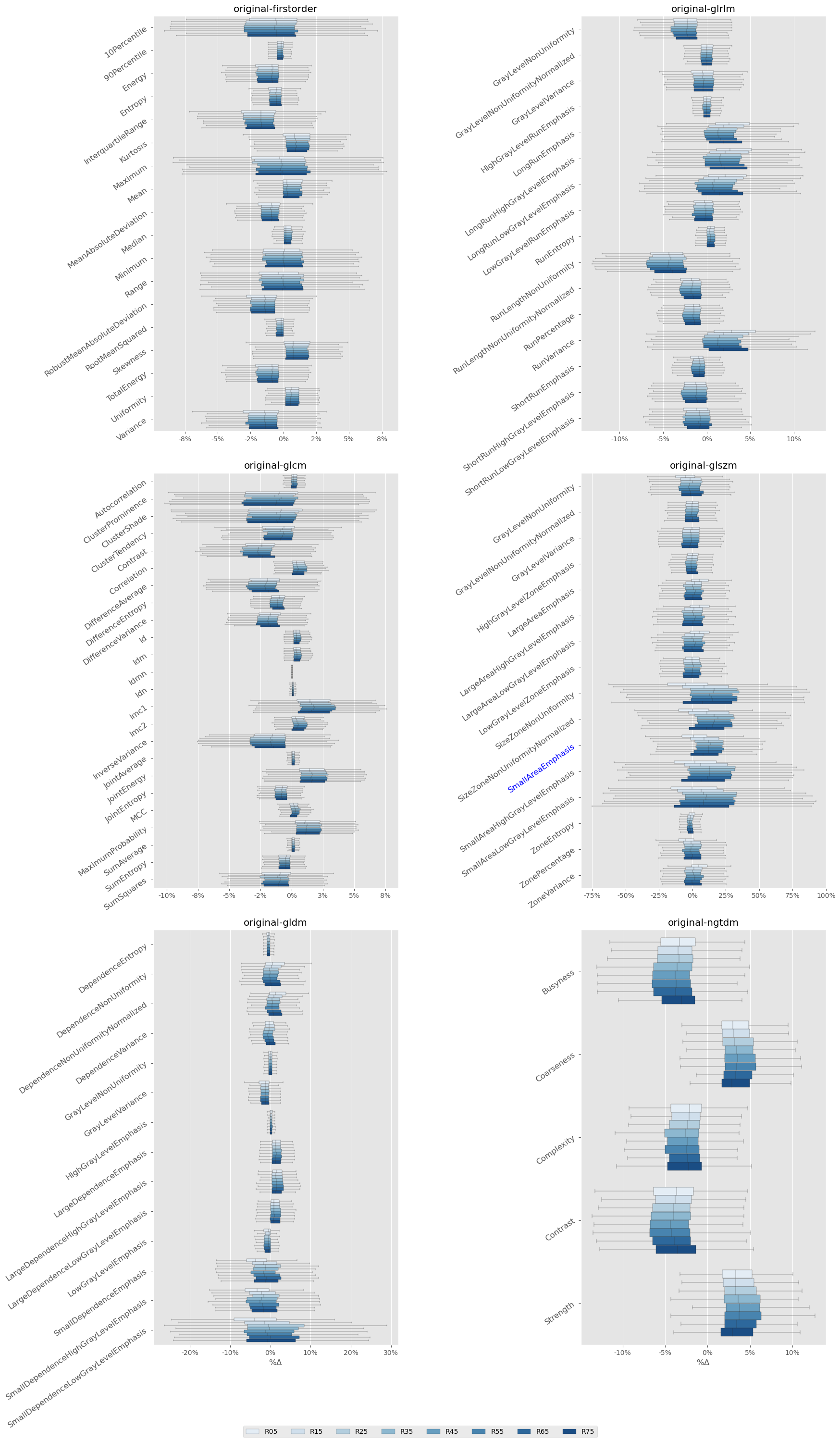


Figure S1 The boxplots visualizing the changes in IQR of %Δ of grouped radiomic features from the original image. The highlight in blue represents the IQR of $\%\Delta$ of that feature were significantly correlated with the degree of rotations applied.


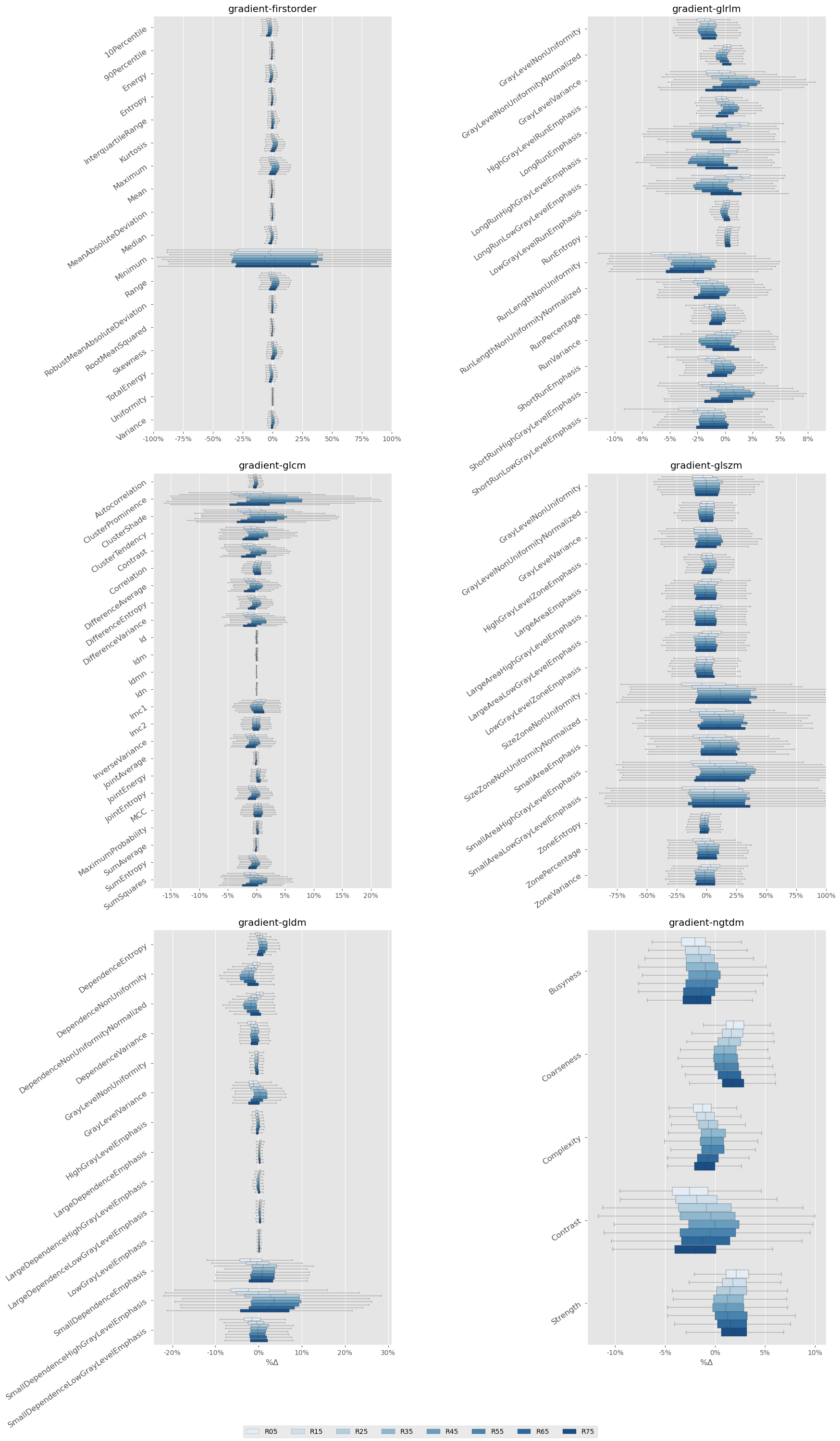


Figure S2 The boxplots visualizing the changes in IQR of %Δ of grouped radiomic features from the imaging filter of “Gradient”.


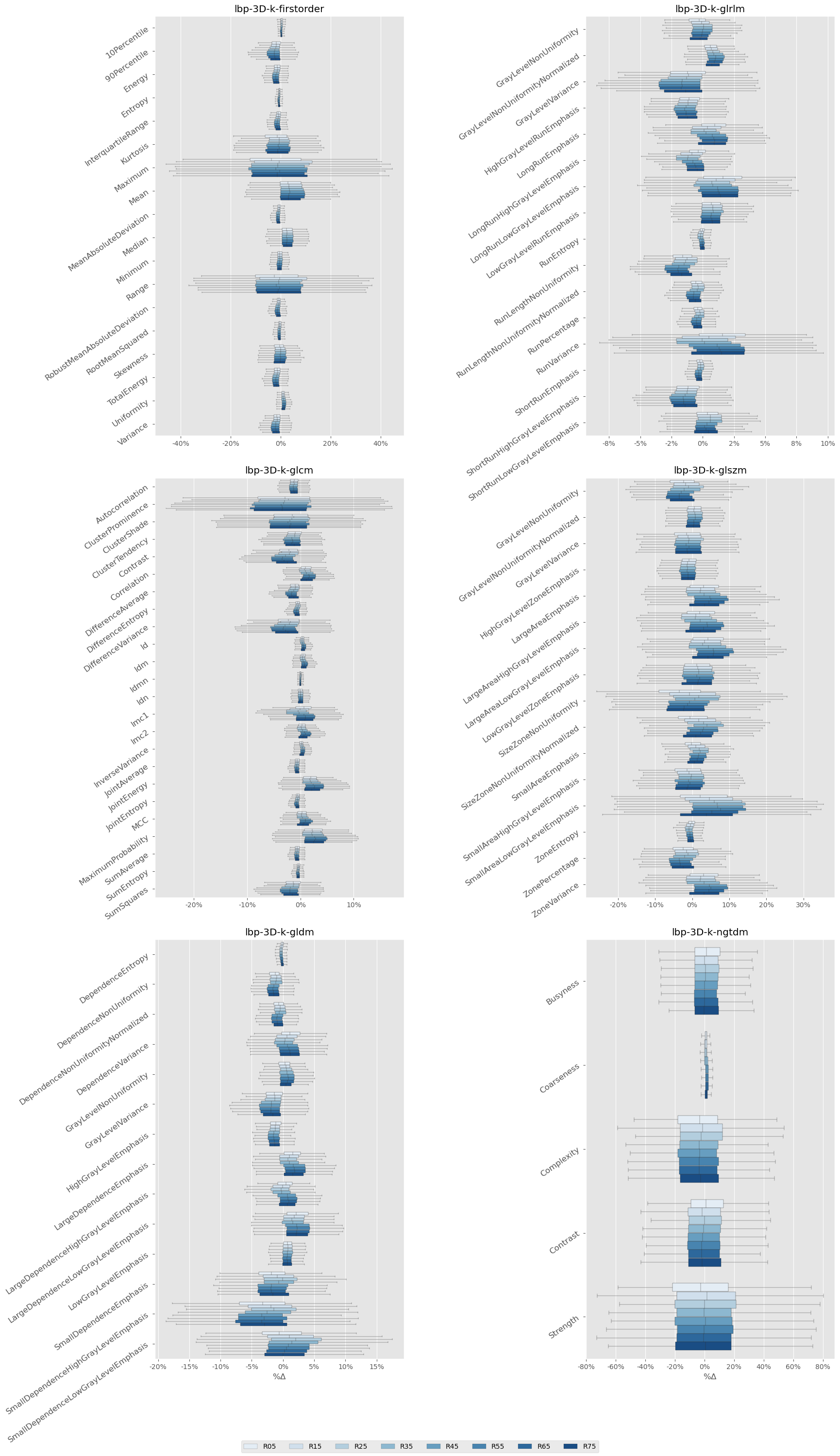


Figure S3 The boxplots visualizing the changes in IQR of %Δ of grouped radiomic features from the imaging filter of “Local Binary Pattern: lbp-3D-k”.


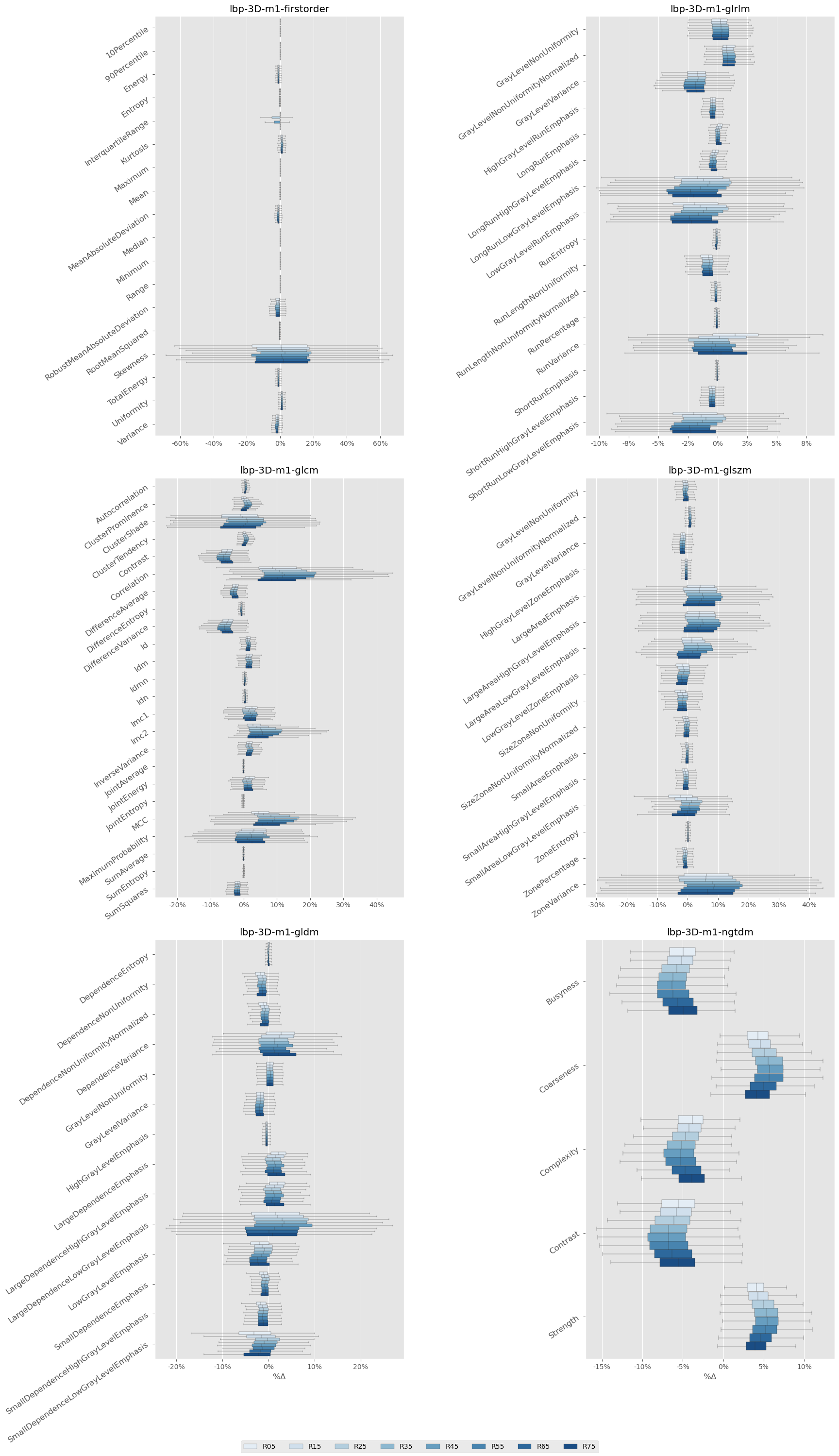


Figure S4 The boxplots visualizing the changes in IQR of %Δ of grouped radiomic features from the imaging filter of “Local Binary Pattern: lbp-3D-m1”.


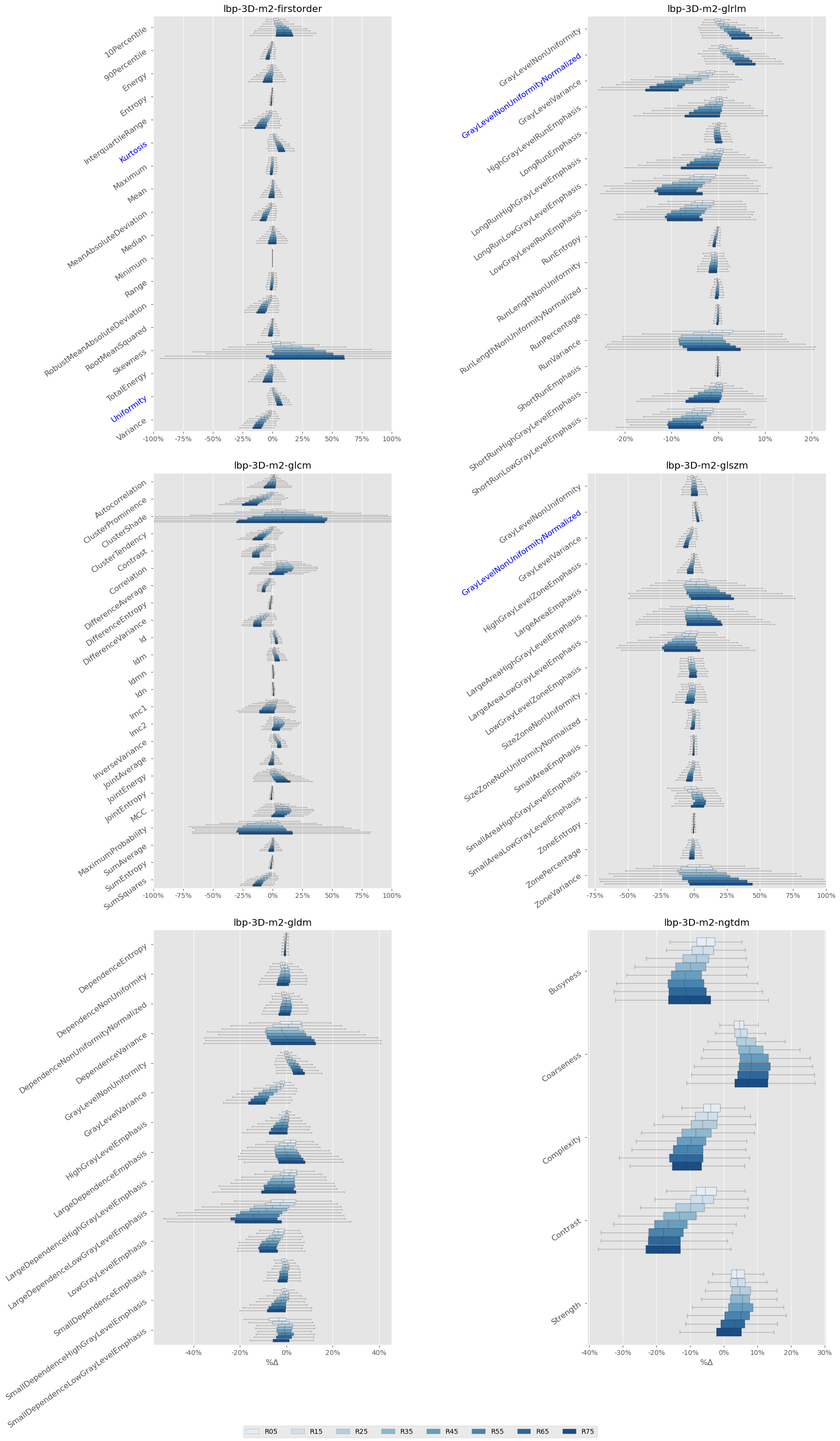


Figure S5 The boxplots visualizing the changes in IQR of %Δ of grouped radiomic features from the imaging filter of “Local Binary Pattern: lbp-3D-m2”. The highlight in blue represents the IQR of $\%\Delta$ of that feature were significantly correlated with the degree of rotations applied.


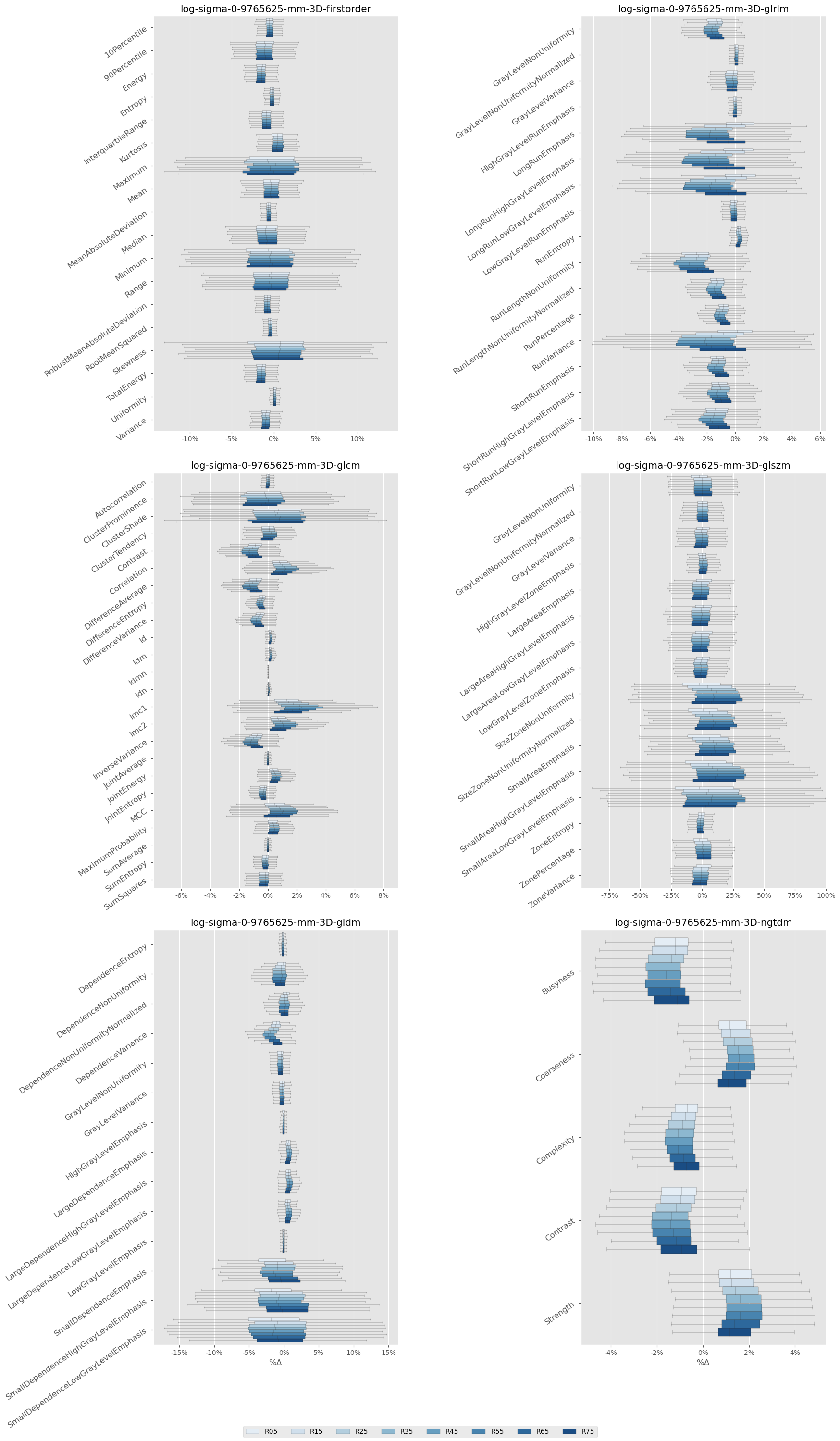


Figure S6 The boxplots visualizing the changes in IQR of %Δ of grouped radiomic features from the imaging filter of “Laplacian of Gassian: log-sigma-0-9765625-mm-3D”.


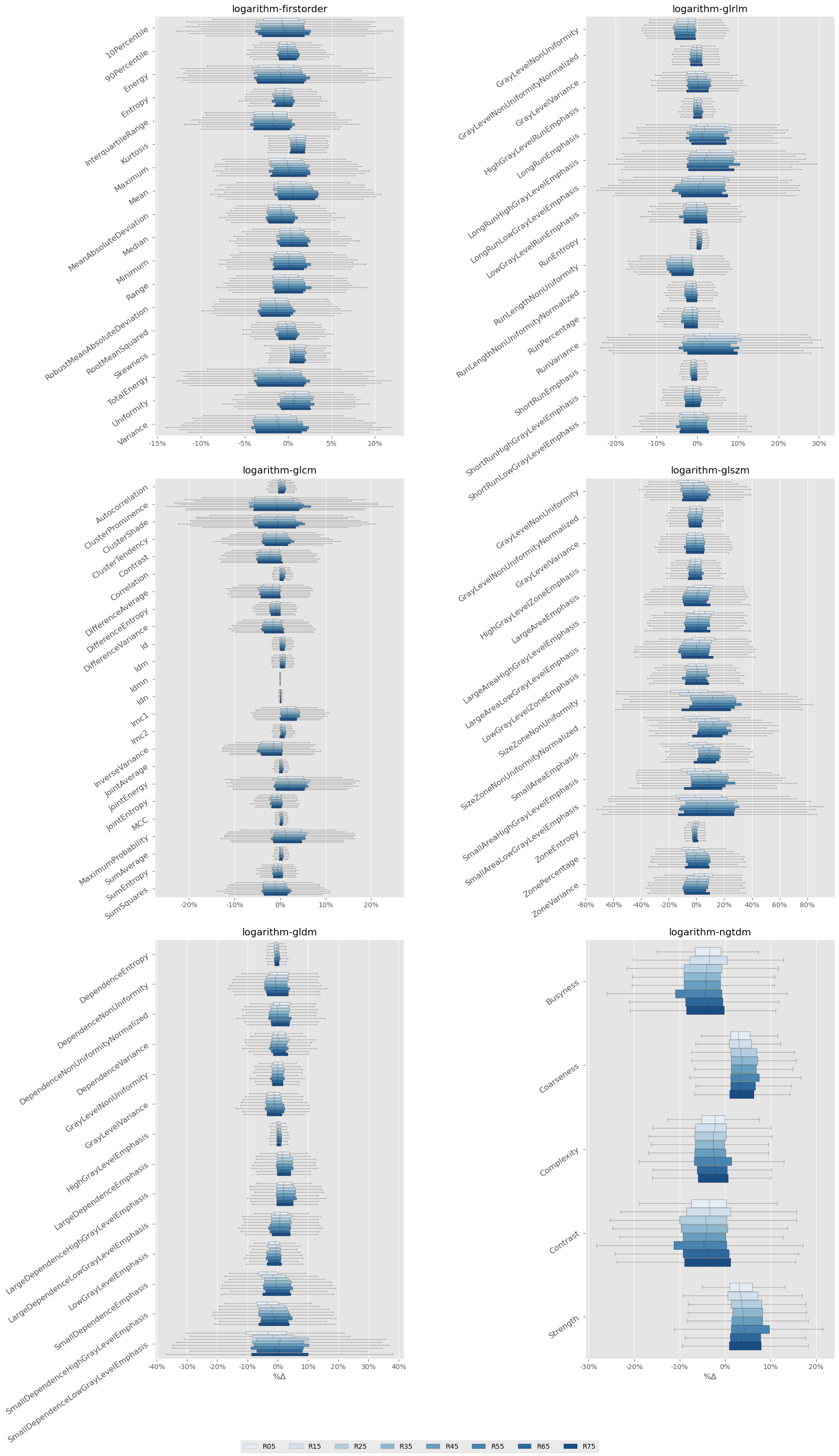


Figure S7 The boxplots visualizing the changes in IQR of %Δ of grouped radiomic features from the imaging filter of “Logarithm”.


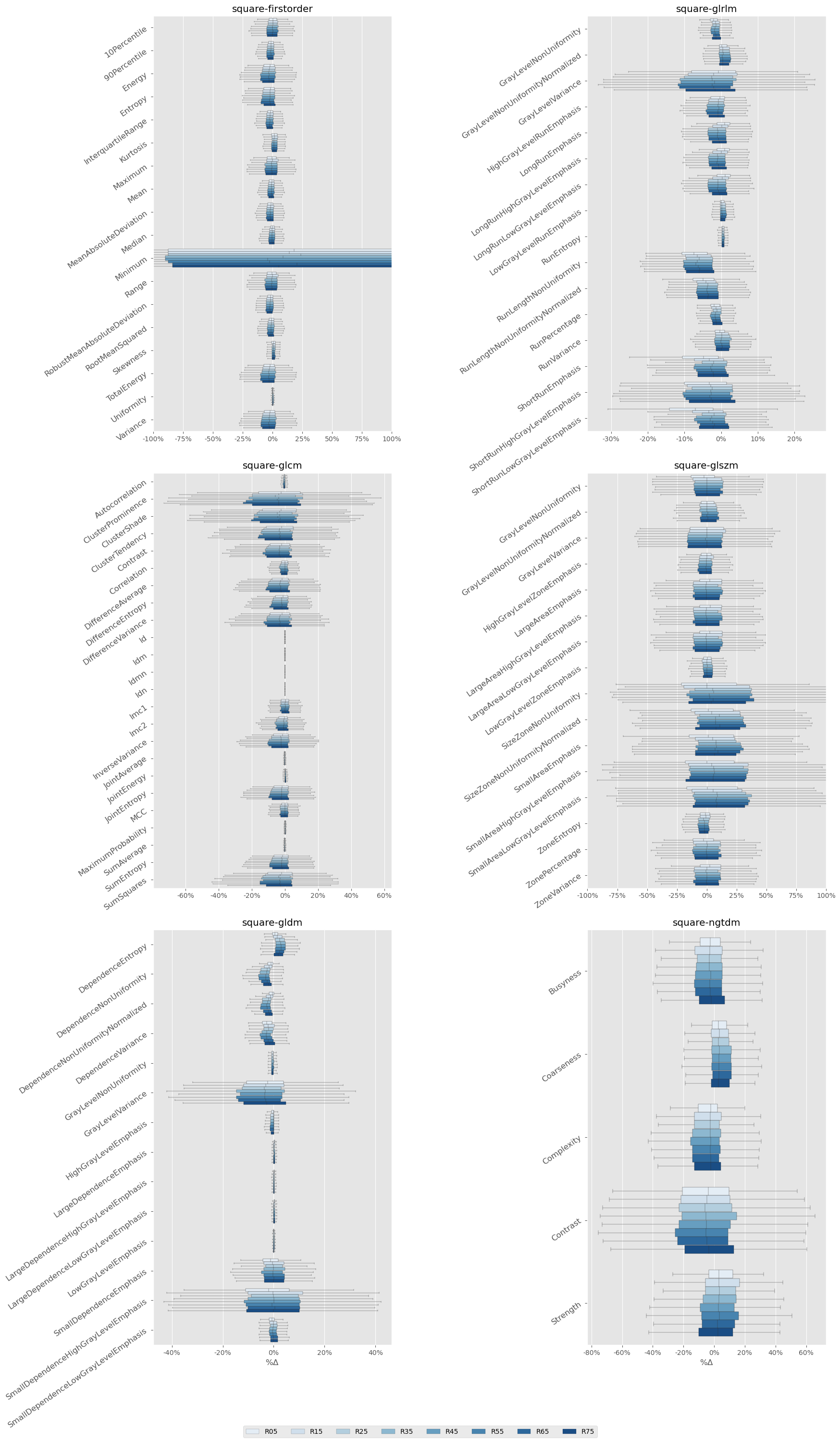


Figure S8 The boxplots visualizing the changes in IQR of %Δ of grouped radiomic features from the imaging filter of “Square”.


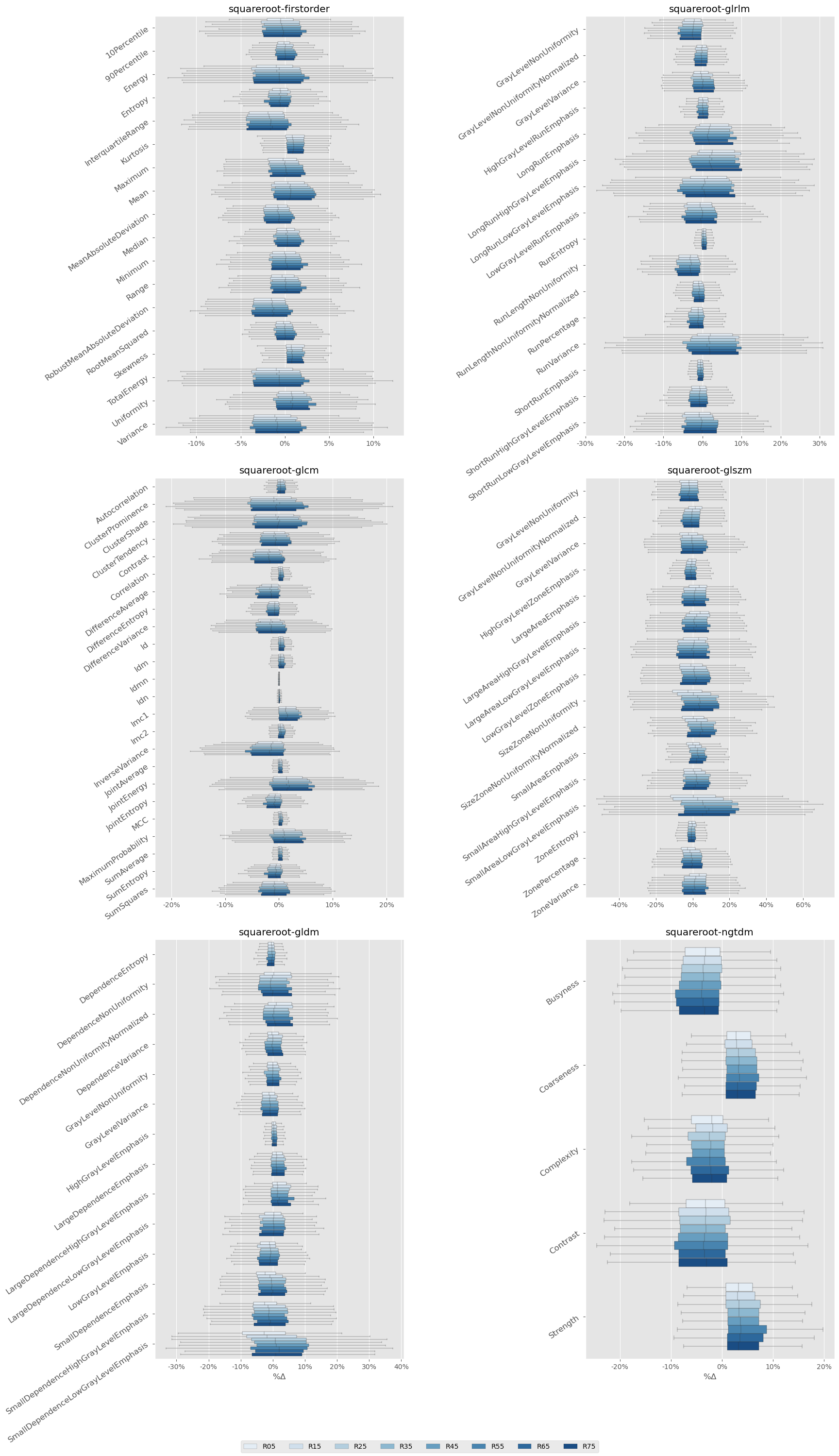


Figure S9 The boxplots visualizing the changes in IQR of %Δ of grouped radiomic features from the imaging filter of “Square Root”.


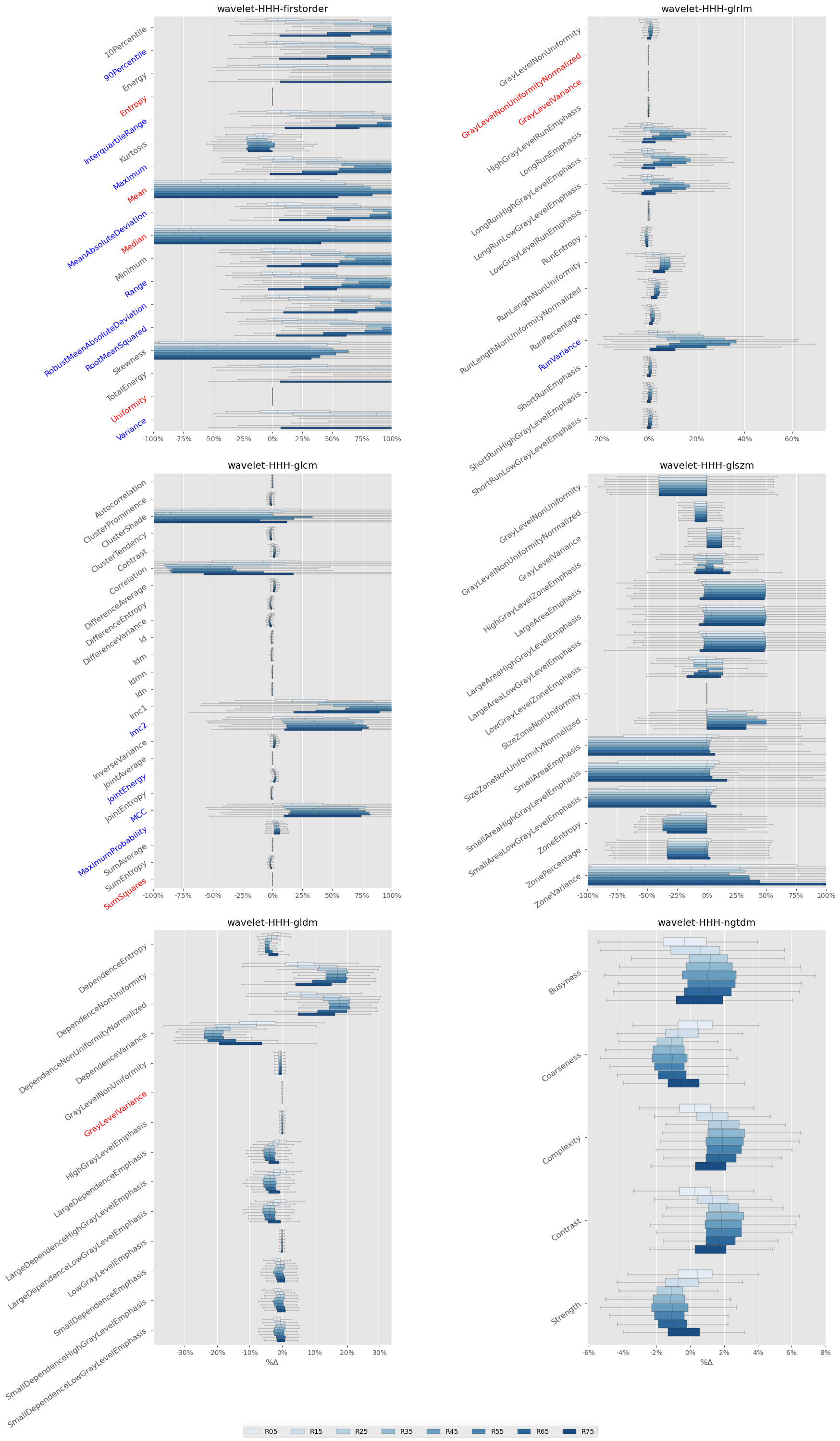


Figure S10 The boxplots visualizing the changes in IQR of %Δ of grouped radiomic features from the imaging filter of “wavelet-HHH”. The highlight in blue represents the IQR of $\%\Delta$ of that feature were significantly correlated with the degree of rotations applied. The highlight in red represents the feature has insignificant variance across all samples.


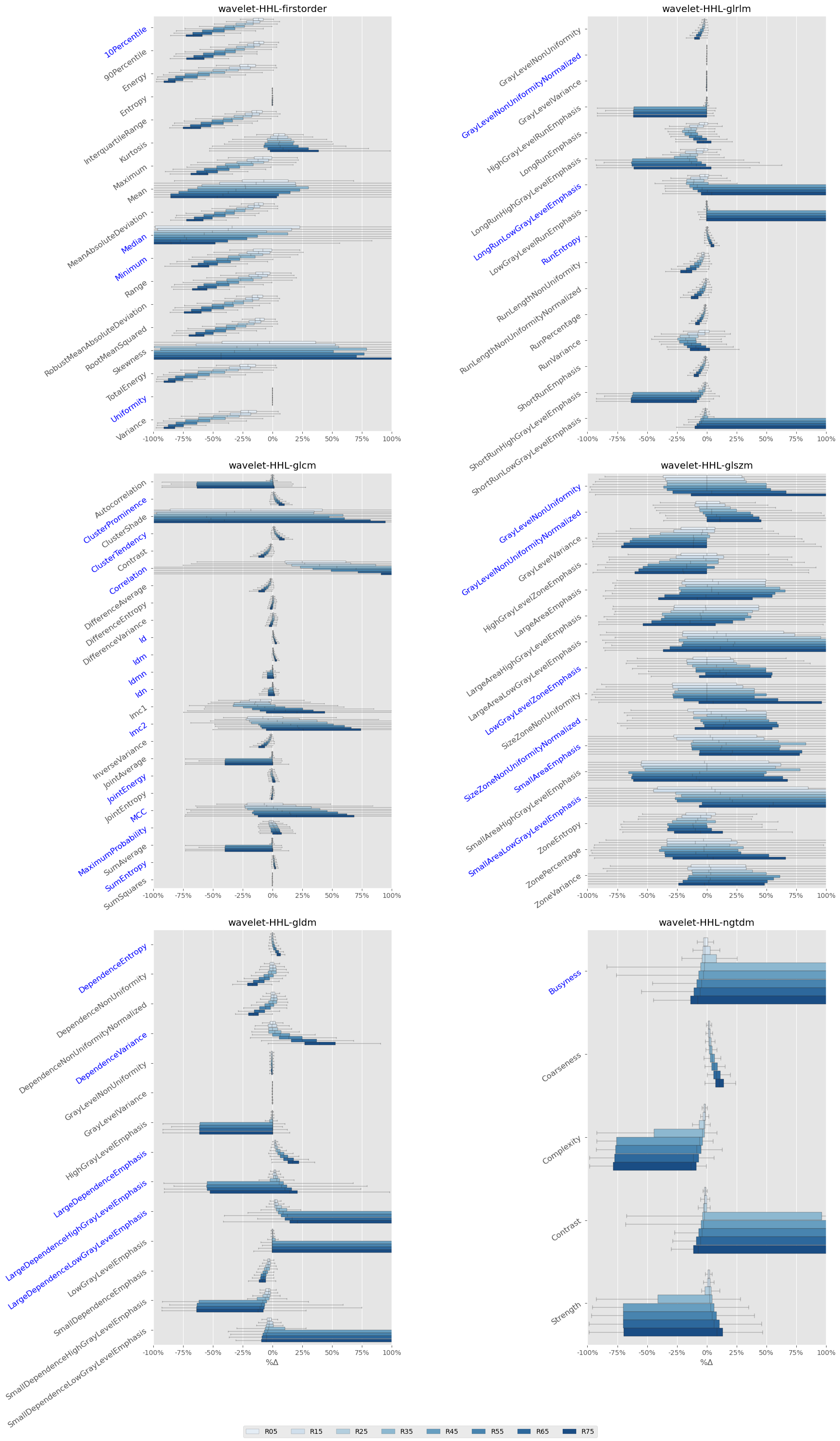


Figure S11 The boxplots visualizing the changes in IQR of %Δ of grouped radiomic features from the imaging filter of “wavelet-HHL”. The highlight in blue represents the IQR of $\%\Delta$ of that feature were significantly correlated with the degree of rotations applied.


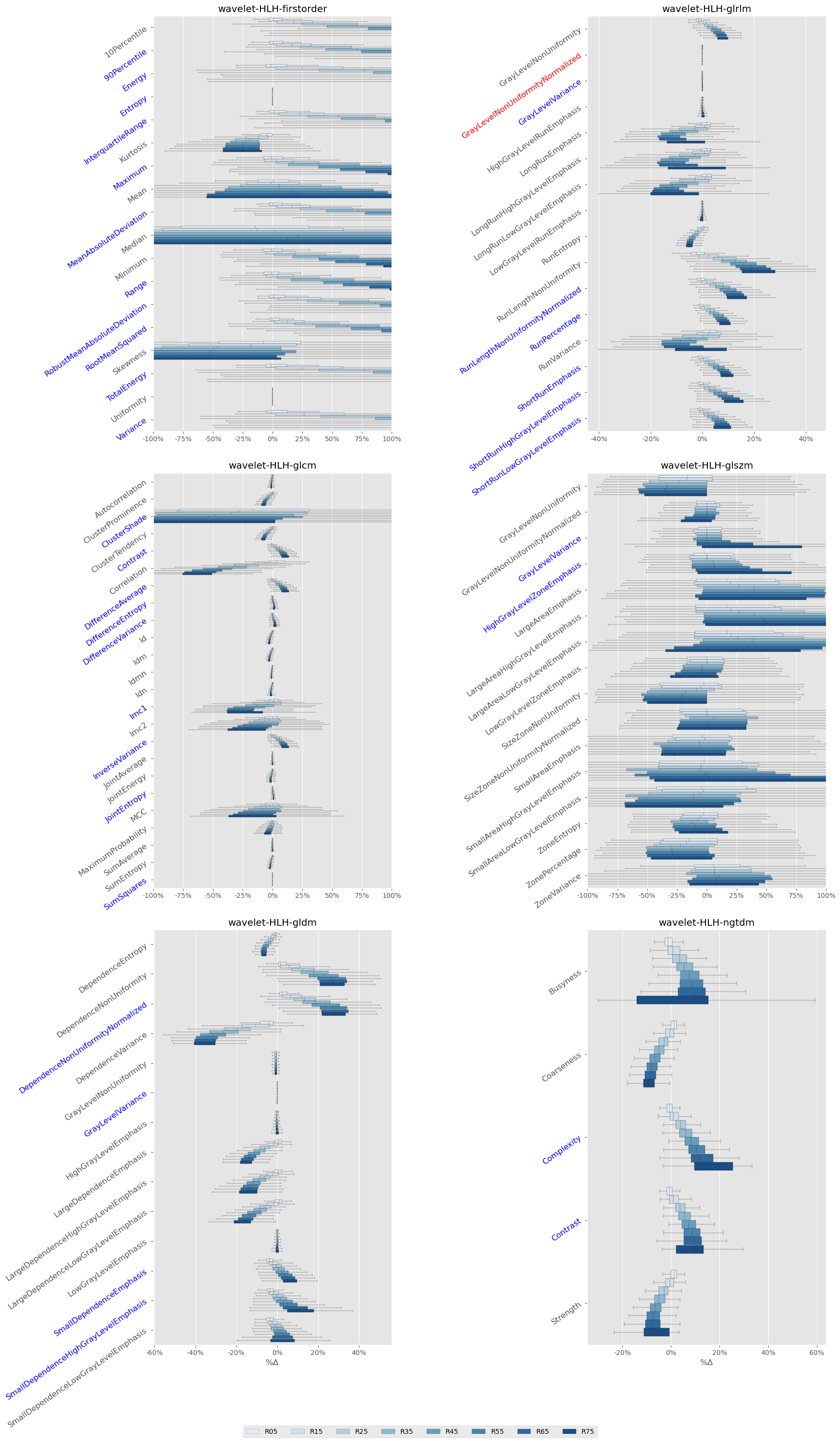


Figure S12 The boxplots visualizing the changes in IQR of %Δ of grouped radiomic features from the imaging filter of “wavelet-HLH”. The highlight in blue represents the IQR of $\%\Delta$ of that feature were significantly correlated with the degree of rotations applied.


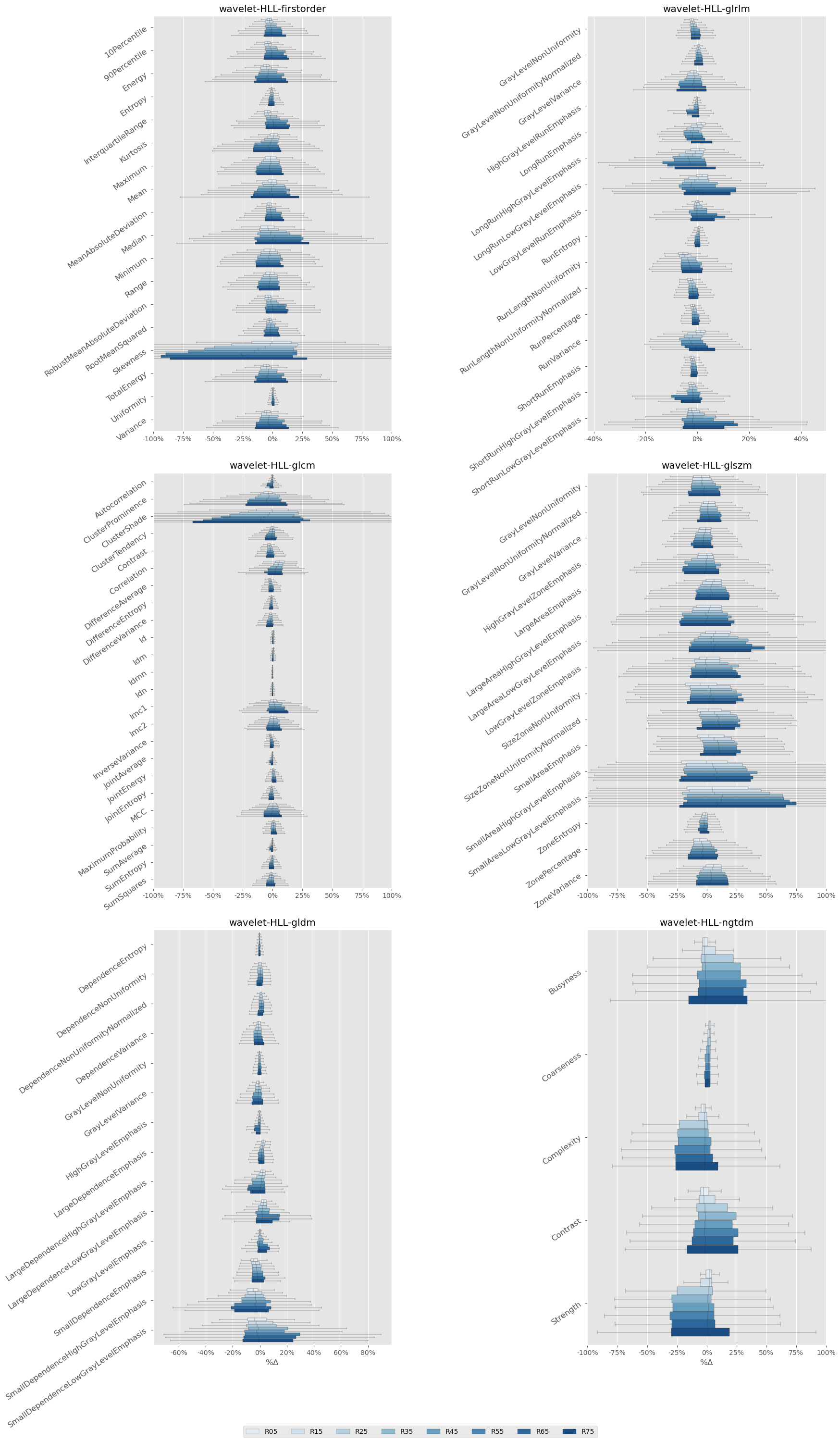


Figure S13 The boxplots visualizing the changes in IQR of %Δ of grouped radiomic features from the imaging filter of “wavelet-HLL”.


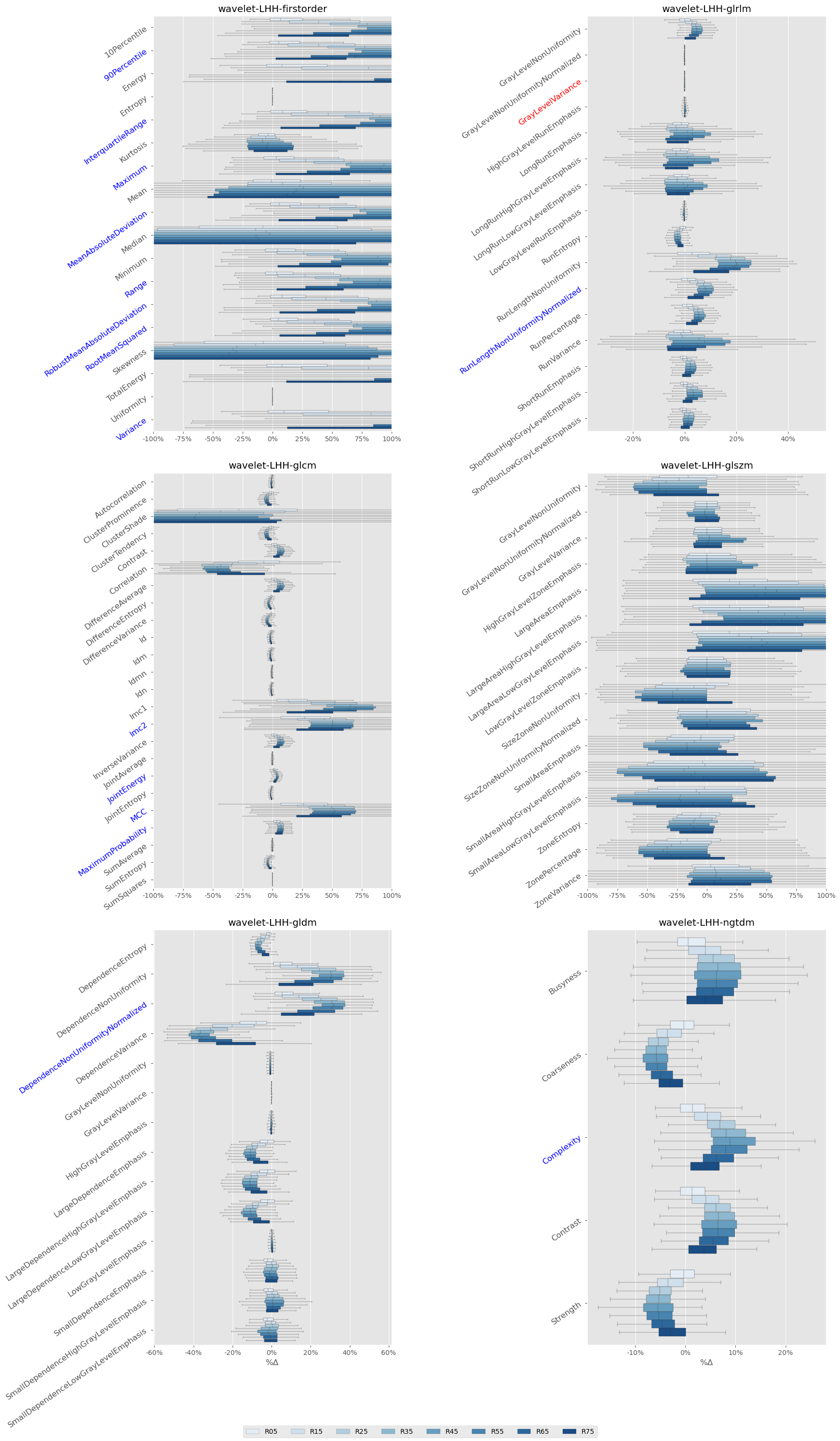


Figure S14 The boxplots visualizing the changes in IQR of %Δ of grouped radiomic features from the imaging filter of “wavelet-LHH”. The highlight in blue represents the IQR of $\%\Delta$ of that feature were significantly correlated with the degree of rotations applied. The highlight in red represents the feature has insignificant variance across all samples.


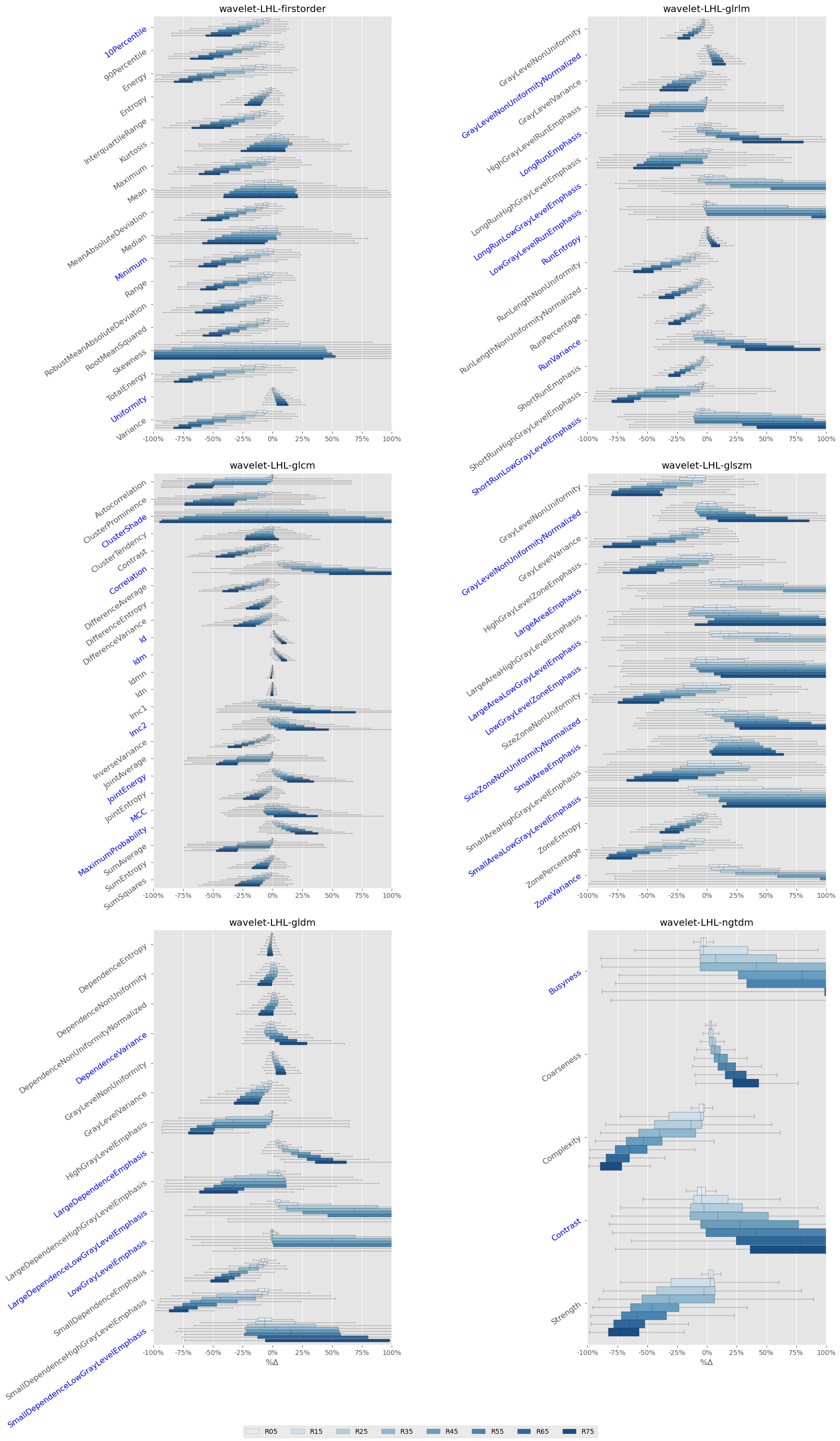


Figure S15 The boxplots visualizing the changes in IQR of %Δ of grouped radiomic features from the imaging filter of “wavelet-LHL”. The highlight in blue represents the IQR of $\%\Delta$ of that feature were significantly correlated with the degree of rotations applied.


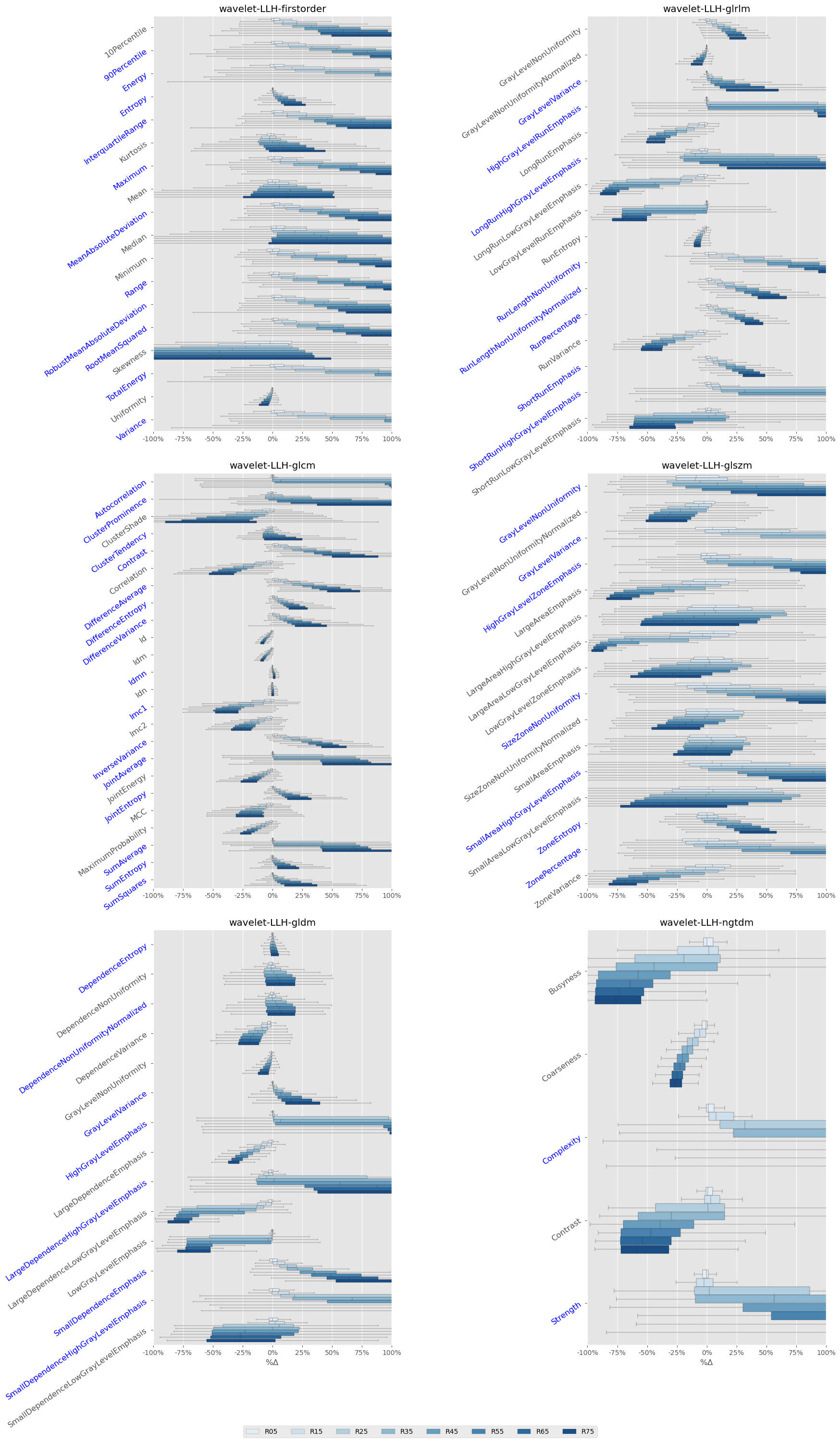


Figure S16 The boxplots visualizing the changes in IQR of %Δ of grouped radiomic features from the imaging filter of “wavelet-LLH”. The highlight in blue represents the IQR of $\%\Delta$ of that feature were significantly correlated with the degree of rotations applied.


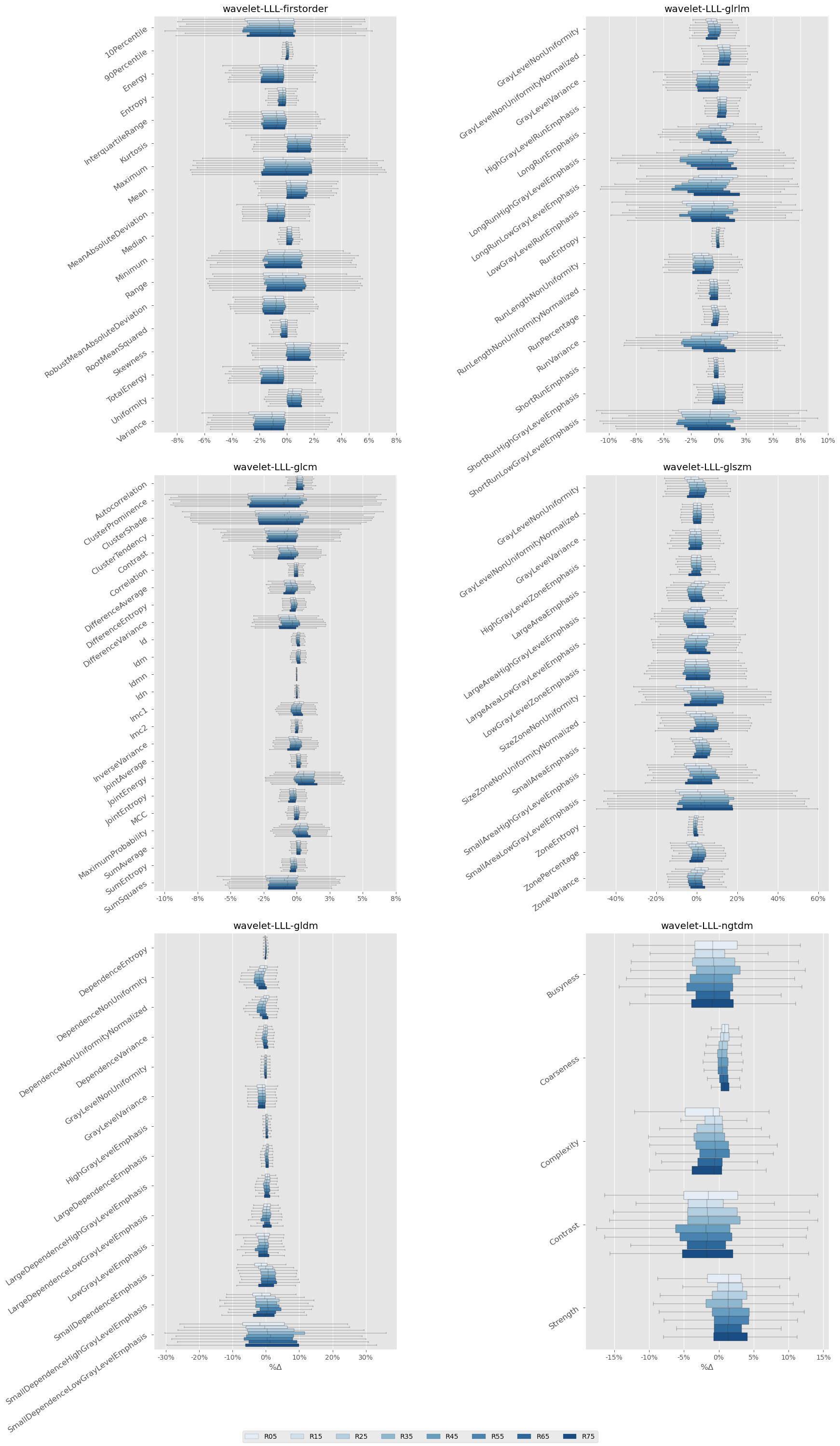


Figure S17 The boxplots visualizing the changes in IQR of %Δ of grouped radiomic features from the imaging filter of “wavelet-LLL”.
