# Supplementary material for: Decoding the Rotation Effect: A Retrospective Analysis of Lesion Orientation and Its Impact on Wavelet-Based Radiomics Feature Extraction and Lung Cancer Classification
Source: J Imaging Inform Med. 2025 May 6;39(1):265–76. doi: 10.1007/s10278-025-01520-8 (PMC12920822; doi:10.1007/s10278-025-01520-8)
Supplement: Supplementary file 4 — Supplementary file4 (DOCX 21 KB) [file 10278_2025_1520_MOESM4_ESM.docx]

Supplementary material – Appendix D: Additional Discussion

## Additional discussion based on boxplots of IQR of $\boldsymbol{\%\Delta}$ of each feature

### Visualizing the IQR of $\boldsymbol{\%}\boldsymbol{\Delta}$ for each imaging filter

Using boxplots, we visualized the changes in IQR of $\%\Delta$ of radiomic features of non-small-cell lung cancer (NSCLC) primary tumor when various degrees of rotation were applied to it. The feature names on the y-axes of the plots are highlighted in blue if its IQR of $\%\Delta$ were significantly correlated with the degree of rotations applied (Spearman’s correlation coefficient ≥ 0.1 and p < .05). They are highlighted in red if it was filtered away by the variance threshold, which indicates they are taking nearly identical values for all patients’ NSCLC primary tumours. Furthermore, to reduce complexity of the boxplots, we plotted the rotations from $R_{0}$ to $R_{80}$ with 10 degree intervals instead of 5. A total of 17 figures (Figure SB1 to Figure SB17) were plotted corresponding to the 17 imaging filters used in this study. They are provided separately together with their legends.

### Extended discussion on observations

In addition to the observations discussed in the main text, three distinct patterns of changes in $\%\Delta$ against the induced rotation were observed in features that exhibit non-trivial correlation as follows: 1) the median and IQR grows with the rotated degree monotonically; 2) the median raises up initially, but then a turning point at 45 degree is seen, with majority of these features showed a relatively stable IQR across different rotation degrees; 3) both the median and IQR jumped up significantly at 45 degree, this pattern is most common in wavelet-filters-based features. Furthermore, we also found the patterns of wavelet-filters to be more drastic when compared to other imaging filters in general. But once again, the LLL component displayed a relatively stable behaviour that is consistent with the observation we reported int the main text.

Interpreting these additional patterns, we expected the first pattern, showing for these features, the deviation in feature value grows monotonically with increasing rotation degree applied intuitively. The second pattern, however, is more interesting, suggesting that there might be unrevealed symmetry in some of the 3D texture with the maximum deviation occurring at 45 degrees from original orientation. The third pattern occurred mainly for WD features. The abrupt increase in $\%\Delta$ at roughly 45 degrees often affects only either the upper or the lower quartile, not the entire distribution. This suggests that for WD features, some information became entirely different for some patients at 45 degrees. It is speculated that the information might have been transferred to another wavelet component whilst information originally in other wavelet components was transferred to it, resulting in such drastically different distribution of $\%\Delta$.
